# Application of Skyline software for detecting prohibited substances in doping control analysis

**DOI:** 10.1371/journal.pone.0295065

**Published:** 2023-12-05

**Authors:** Hyeon-Jeong Lee, Mijin Jeon, Yoondam Seo, Inseon Kang, Wooyeon Jeong, Junghyun Son, Eugene C. Yi, Hophil Min

**Affiliations:** 1 Doping Control Center, Korea Institute of Science and Technology, Seongbuk-gu, Seoul, Republic of Korea; 2 Department of Molecular Medicine and Biopharmaceutical Sciences, Graduate School of Convergence Science and Technology, Seoul National University, Yeongeon-dong, Jongno-gu, Seoul, Republic of Korea; University of California Riverside, UNITED STATES

## Abstract

As the number of prohibited drugs has been progressively increasing and analytical methods for detecting such substances are renewed continuously for doping control, the need for more sensitive and accurate doping analysis has increased. To address the urgent need for high throughput and accurate analysis, liquid chromatography with tandem mass spectrometry is actively utilized in case of most of the newly designated prohibited substances. However, because all mass spectrometer vendors provide data processing software that is incapable of handling other instrumental data, it is difficult to cover all doping analysis procedures, from method development to result reporting, on one platform. Skyline is an open-source and vendor-neutral software program invented for the method development and data processing of targeted proteomics. Recently, the utilization of Skyline has been expanding for the quantitative analysis of small molecules and lipids. Herein, we demonstrated Skyline as a simple platform for unifying overall doping control, including the optimization of analytical methods, monitoring of data quality, discovery of suspected doping samples, and validation of analytical methods for detecting newly prohibited substances. For method optimization, we selected the optimal collision energies for 339 prohibited substances. Notably, 195 substances exhibited a signal intensity increase of >110% compared with the signal intensity of the original collision energy. All data related to method validation and quantitative analysis were efficiently visualized, extracted, or calculated using Skyline. Moreover, a comparison of the time consumed and the number of suspicious samples screened in the initial test procedure highlighted the advantages of using Skyline over the commercially available software TraceFinder in doping control.

## Introduction

Remarkable developments in mass spectrometry (MS) instrumentation have enabled rapid and sensitive screening of hundreds of chemicals using targeted selected reaction monitoring (SRM) methods [1]. To analyze the compounds of interest, software programs helping users to develop and optimize SRM analysis have been available in proteomics for over a decade [2–4]. Skyline, an open-source software program launched for targeted proteomics, has proven to be a reliable, flexible, and widely used program for targeted SRM–MS analysis [5]. As Skyline has emerged and acquired high credibility among users, variable approaches such as those for small-molecule platforms including metabolomics [6] and lipidomics [7] and the quantification of data-dependent acquisition data are suggested [4, 8]. Although several attempts have been made to use Skyline in many other fields, the application of this software program in doping control to perform the target analysis of various types of compounds including peptides and small molecules has not yet been reported.

Every year, the World Anti-Doping Agency (WADA) publishes an updated list of prohibited substances, which has been progressively expanded from small molecule to peptide hormones with medium molecular weight such as insulin, synacthen, and IGF1 analogs [9]. The workload of doping control has increased because the analytical methods for newly designated prohibited drugs have to be designed and validated after the annual renewal and the minimum required performance levels (MRPLs) of analytical methods have been lowered to improve the detectability of drug analysis [9, 10]. As the workload involved in sports drug testing is steadily increasing, mitigating laborious procedures to review all data while maintaining the highest possible sensitivity and detectability of analysis during analysis is highly required.

So far, doping control analysis, including data screening and method development, has been accomplished using various commercially available software programs. The Excel-based reporter tool named as TraceFinder was introduced in doping control in a previous study [11], processing the analytical results using the reporter layout that contains extracted ion chromatograms of target ions and confirming ions. Like TraceFinder from Thermo, each MS vendor, including Waters, Sciex, and Agilent, offers their vendor-dependent data processing software programs such as UNIFI [12], Sciex OS, and MassHunter [13]. However, finding a commercially available program that can efficiently and effectively handle all the necessary steps involved in doping control analysis within a single software platform is challenging. These steps include method development and optimization, data processing, and quality control monitoring. In addition, the use of multiple commercial software programs for each step may introduce inconsistencies and errors in data analysis. Therefore, a unified and comprehensive software is required to address this major challenge and enhance the efficiency, accuracy, and reliability of the doping control process. Skyline supports method development—from method validation to optimization—and data processing using raw files from six major instrument vendors; Agilent, Bruker, Sciex, Shimadzu, Waters, and Thermo [14]. Moreover, Skyline provides comprehensive visualization and sophisticated method-development options such as retention-time filtering, peak integration, collision energy (CE) optimization, and longitudinal detection–quality monitoring, which make the review of large-scale data easy and efficient.

Generally, the sensitivity of SRM is considerably affected by CE because excessive or insufficient CE reduces the signal intensity reaching to the detector [15]. Empirically, CE optimization has been performed through direct infusion; however, this process is considerably time and resource intensive and requires high purity standards [16]. Therefore, determining the best CE for hundreds of targets by the direct infusion is laborious and inefficient. In contrast, Skyline uses data obtained during actual analyses, making it possible to achieve CE optimization considering matrix effects on analyte fragmentation. In addition, Skyline automatically calculates ranges of CE sets based on the analyst’s setting and sums the peak area observed over replicates to enhance the accuracy of the optimal CE [16]. In proteomics, Skyline suggests the primary CE value for doubly- or triply-charged peptides using instrument-specific and built-in linear equations from the precursor mass-to-charge ratio (m/z) [16, 17]. However, a linear equation for CE prediction during small-molecule analysis is not supported so far. In this study, we optimized the CEs of 339 small molecules using Skyline.

In addition to enhancing the sensitivity of analytes, maintaining the detection quality—which certifies that the method and the analytical instrument produce an accurate and precise result—is one of the most important requirements to ascertain the credibility of data. Control charts monitoring the results of an internal reference provide an internal quality system that enables the evaluation of the longitudinal quality performance [18]. In doping control, the use of internal standards (ISTDs) is a general strategy for internal quality control by monitoring the internal standard peak in each sample of the batches. This enables the analyst to manage the substantial possible error associated with the injector, column, mobile phase, or detector [19]. However, archiving detector signals of internal standards for years is cumbersome. Skyline offers an automated quality control process for monitoring the detection sensitivity of internal standards. Panorama is a web-based module for storing, sharing, and disseminating results imported in Skyline [20, 21]. Along with storing and disseminating Skyline results through AutoQC, newly acquired data files were automatically analyzed using Skyline to monitor for system suitability and were instantly uploaded into a designated folder in Panorama. The imported results were statistically analyzed using several QC metrics and visualized using a Levey–Jennings chart [22].

Overall, in this study, we envision the application of Skyline to establish a systematic workflow of doping control including method optimization, sample screening, longitudinal detection–quality monitoring, and method validation. For future use, we recommend that other doping labs should use the Skyline platform to improve coherent analysis within the doping society.

## Materials and method

### Reagents and materials

Citric acid monohydrate and sodium citrate tribasic dihydrate were purchased from Sigma-Aldrich (St. Louis, MO, USA). β-Glucuronidase from *Escherichia coli* was produced by Roche Diagnostics (Mannheim, Germany). Methyl alcohol was obtained from J.T. baker (Radnor, PA, USA). Formic acid and phosphoric acid were supplied by Wako (Richmond, VA, USA) and Yakuri (Kyoto, Japan). Deionized water was acquired from a Millipore water purifier (Burlington, MA, USA). The mixed‐mode weak cation exchange (WCX) 3cc cartridge from Waters (Milford, MA, USA) was used for the solid phase extraction.

### Sample preparation

#### Ethics statement

This study was reviewed and approved by the institutional review board (IRB) at the Korea Institute of Science and Technology (KIST-202304-BR-005 and KIST-202306-HR-004). The collection of urine samples from volunteer athletes followed the Guidelines for Urine Sample Collection provided by the WADA. Subsequently, after conducting the doping test, the negative urine samples that verified the absence of prohibited drugs were combined and used as blank urine samples. Additionally, to compare the doping screening efficiency between Skyline software and commercial alternatives, a study was conducted involving six volunteers who possessed doping control experience and the ability to interpret MS results. Based on their level of doping control proficiency, the volunteers were divided into two groups: early and advanced groups. All volunteers were provided with both verbal and written information about the study purpose, methods, and implications for them and for the field of study before obtaining their written consent to participate.

#### Qualification analysis

A urine sample was prepared for qualitative analysis according to the procedure reported a previous study [23]. Briefly, a 10-μL aliquot of the ISTD solution was added to each urine sample (2 mL). A 1-mL aliquot of citrate buffer (0.1 mol/L; pH 6.0) was used to adjust the sample pH to 6.0. Then, for enzyme hydrolysis, a 50-μL sample of β‐glucuronidase was added and incubated at 55°C for 1 h using a water bath. After the hydrolysis of the sample, a 100-μL aliquot of 4% phosphoric acid was added and then the sample was centrifuged for 5 min at 3000 g. Mixed‐mode WCX cartridges (60 mg; Waters, Milford, MA, USA) were activated before sample loading using 2-mL aliquots of methanol and water. After the activation of the cartridge, the sample was loaded on the activated catridge and cleaned with 2 mL of water and with 2 mL of methanol. Then, the target compounds were eluted with a 1-mL aliquot of 2% ammonia and 1.2% formic acid in methanol. Eluents were evaporated using a N_2_ stream at 50°C, reconstituted in 200 μL of 1% formic acid in 5% methanol, and then 10-μL aliquots of the resulting solution were injected into the liquid chromatography with tandem MS (LC-MS/MS) system.

#### Quantitative analysis

The urine samples for quantitative analysis were prepared by slightly modifying the procedure reported in a previous study [24]. Subsequently, 20-μL urine or a calibrator was prepared, with the nominal concentrations of 0, 5, 7.5, 9, 10.5, 14, 17.5, and 20 μg/mL. An isotope of ephedrine (d3-ephedrine) was used as the internal standard at a final concentration of 5 μg/mL. After the addition of the internal standard and 210 μL of 0.1% formic acid in 2% acetonitrile, the diluted urine was vortexed and centrifuged at 16000 g for 10 min. The supernatant was injected into LC-MS/MS.

### LC-MS/MS condition

#### Qualification analysis

A Vanquish liquid chromatographic system coupled with a TSQ Altis mass spectrometer (Thermo Scientific, CA, USA) was used for LC-MS/MS analysis. Chromatographic separation was achieved using a Kinetex C18 100 Å LC column (100 mm × 2.1 mm; 2.6 μm; Phenomenex, CA, USA). The temperatures of the autosampler and the column oven were 10°C and 35°C, respectively. Solvent A was water and solvent B was methanol. Both mobile phases included 0.1% formic acid. The separation procedure, which lasted for 10 min, was conducted at a flow rate of 0.5 mL/min. Initially, the gradient started at 2% B, stayed constant for 5 min, followed by a linear increase to 95% B in 8 min, and then stayed constant again at 95% B for 0.5 min, and then returned to 2% for 1 min. The injection volume was 10 μL.

A mass spectrometer equipped with an H-electrospray ionization (ESI) ion source was used for ESI. The detailed ESI source settings are described below. The positive and negative ion spray voltages were 4500 and 3500 V, respectively, the sheath gas was 60 arbitrary units, the aux gas was 15 arbitrary units, the sweep gas was 1 arbitrary unit, the ion transfer–tube temperature was 320°C, and the vaporizer temperature was 340°C. A scheduled SRM method with Q1 and Q3 resolutions set at 0.7 and polarity switching was employed to acquire data. For most of the compounds, the detection window was 1 min, the target cycle time was 0.4 s, and the dwell time was at least 7 ms per SRM experiment.

#### Quantitative analysis

A UFLC XR series high-performance LC system (Shimadzu, Japan) coupled with a TSQ Ultra triple quadrupole mass spectrometer (Thermo Scientific, CA, USA) was used for the quantitative analysis. Chromatographic separation was performed on an ACE C18 column (100 mm × 2.1 mm; 5 μm; Advanced chromatography technologies). The temperatures of the autosampler and column oven were 10°C and 35°C, respectively. The mobile phase comprised 0.1% aqueous formic acid as solvent A and 0.1% formic acid in acetonitrile as solvent B. The LC gradient conditions were achieved at a flow rate of 0.4 mL/min, and the initial percentage of 2% B was increased to 7% B in 0.09 min, and then raised to 16% B in 5.7 min. To wash the remaining compound, the percentage of solvent B was increased to 95% in 0.2 min and maintained for 0.6 min. Subsequently, re-equilibration to 2% B was conducted at 6.6 min and maintained for 8 min, ending the overall run at 8 min. The injection volume was 4 μL.

The TSQ Ultra triple quadrupole equipped with an ESI source was used in a positive ionization mode. The positive-ion spray voltage was 4500 V, and the sheath-gas pressure was 60 arbitrary units. The capillary and vaporizer temperatures were set to 320°C and 340°C, respectively. The Q1 and Q3 resolutions were set to 0.7, and for the collision gas, the pressure was maintained at 0.19 Pa and the energy was set to 20 eV. The transitions for ephedrine and d3-ephedrine were from 166 to 133 m/z and 169 to 117 m/z, respectively.

### Method validation for newly prohibited materials

To validate the analytical methods, selectivity, reliability of detection, linearity, intraday and interday precision, sample stability, the limit of detection (LOD), the limit of identification (LOI), carryover, matrix effect, and recovery were used as method-validation parameters. All method-validation parameters were used to properly analyze the MRPLs; 100 ng/mL, the concentration declared in the WADA technical document; TD2019MRPL. Each parameter was evaluated according to the ISO/IEC 17025 guidelines and WADA laboratory technical note on analytical method validation for doping control analysis.

### Selectivity

Selectivity was evaluated for verifying the absence of potential interferences toward a target analyte on the blank sample. For this, 10 blank urine samples from different people were analyzed.

### Reliability of detection

The consistency of results was assessed in the presence of low levels of target compounds using the MRPL of each compound. A total of ten representative samples were analyzed dividing in two batches.

### Linearity

Linearity of the calibration curves was determined using R-squared score (R^2^) based on the ratio of the analyte peak areas to the ISTD peak areas and the concentrations of the standards from calibration curves. Seven concentration points, i.e., 20%, 50%, 100%, 250%, 500%, 1000%, and 2500%, of MRPL were analyzed in triplicates.

### Intraday and interday precision

Using the same operating system, a batch containing six MRPL samples was analyzed within a day for intra-assay precision. Three batches containing six samples each were extracted and analyzed over one-day interval for interassay precision. Coefficients of variation (CVs) were used for the evaluation.

### Sample extract stability

To evaluate the stability of target compounds within the matrix component, 11 MRPL urine samples were analyzed again 2, 3, 7, 14, and 21 days after the first analysis.

### LOD and LOI

For evaluating LOD, 50 samples including concentrations of 1%, 2%, 10%, 20%, and 50% MRPL were analyzed and the lowest concentration of the analyte with a signal-to-noise ratio of >3 was chosen as the LOD. Further, the concentration that ensures the criteria of the maximum tolerance windows for relative abundance from WADA TD2021IDCR was chosen as the LOI. The relative abundance of three transitions in MRPL was used as the reference for the relative abundance of three transitions of LOI level.

### Carryover

Carryover of the method was investigated by confirming the second injected blank after the 400% MRPL sample has no observation of any analytes.

### Matrix effect and recovery

The matrix effect of target compounds was evaluated by comparing standard fortified blank urine samples and pure standard mixtures in triplicates. The formula for calculating the matrix effect is ME% = (peak area of spiked standards after elution/peak area of standards) × 100. If a value exceeded 100%, standard ionization was enhanced by the matrix; otherwise, standard ionization was suppressed. The recovery of target compounds was calculated using two types of standard fortified samples in triplicates. The standard was spiked before and after sample preparation of blank urine, and the peak areas of them were compared. The formula for recovery is R% = (peak area of spiked standards before elution/peak area of spiked standards after elution) × 100.

## Results and discussion

Sports doping analyses include various processes such as method validation, result of quality control, and suspicious doping–sample screening. In this study, various cases were applied to determine if the overall doping process could be integrated using Skyline. All acquired raw data were processed and visualized using Skyline version 21.1.0.146 (University of Washington, UW, USA). The workflow detailing the use of Skyline for doping data screening, quantitative analysis, and longitudinal detection–quality-control monitoring using the online data repository called PanoramaWeb and Skyline is described in the supporting information (S1–S2 Texts).

### CE optimization of prohibited materials

Each prohibited chemical was measured based on its unique transition, which has been routinely used for doping control. The CEs for transitions were used as the reference CEs. For 12 newly designated prohibited compounds, the direct infusion was used to acquire their proper ionization mode and ideal CEs and the best transitions of each compound. The most efficient CE for three transitions each for eight compounds was used to confirm the CE optimization of Skyline.

**Fig 1:**
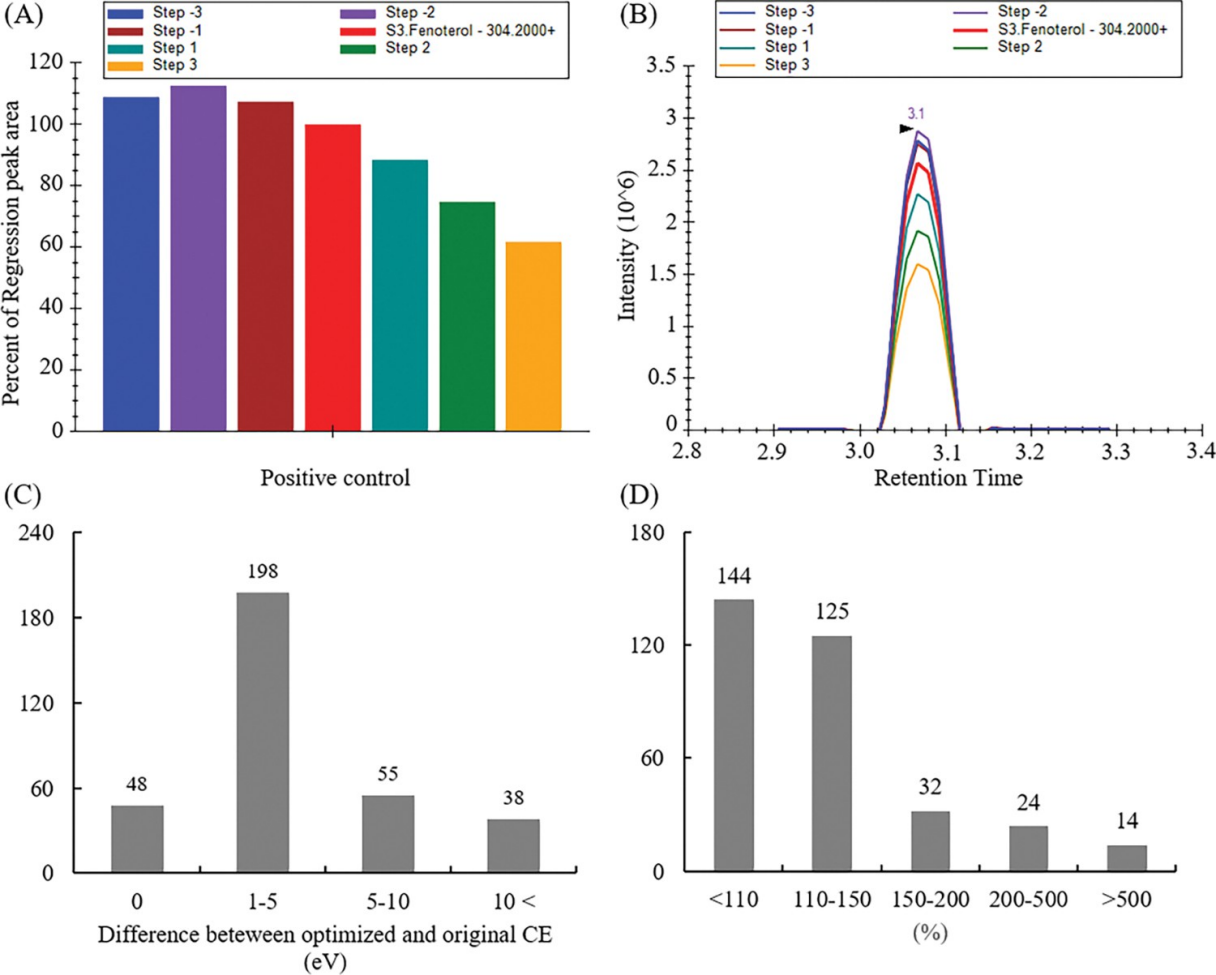
CE profile of fenoterol. (A) Peak-area bar graph indicating seven near-replicate targets for the transition analyzed with different CEs. (B) Corresponding chromatogram showing detection changes according to the modified CE. The bar graph and chromatogram were produced by Skyline. Each CE shows different peak intensities, and these are compared using a bar graph and a chromatogram. The red median bar and line represents the transition observed at its reference CE. (C) Number of transitions exhibiting CE differences after optimization. (D) Number of transitions that increased the detection efficiency using the Skyline CE optimization tool. (C) and (D) were generated by the comparison between optimized CE and reference CE data.

The CE optimization using Skyline proceeded in two steps. In the first step, the CE value with the highest intensity was selected among seven CEs; step size = 3 eV and step count = 3. The median value of stepping was the reference CE in each transition. After that, in the second step, the best CE was selected from seven CEs; step size = 1 eV and step count = 3 based on the initially optimized CE. From the obtained optimization results, we determine the best CE for each compound. To know the exact efficiency of CE optimization achieved by Skyline automatic screening, we analyzed three replicated samples based on two methods that applied original and optimized transition settings, respectively. Fig 1A and 1B verify that the highest intensity of fenoterol (SRM transition from 304.2 to 107.2 m/z) was produced at −6 eV of CE compared with the original CE used in our laboratory during the initial optimization. Thereafter, in the second step, the best CE was selected as 28 eV, which is 7 eV lower than the original CE. Absolute gaps between the original and optimized CE are observed in Fig 1C. More than half of the transitions were modified with minor adjustments (changes between 1 and 5 eV). Ninety-three transitions exhibited an optimal CE switch of >5 eV. The ion intensity–increase ratio was calculated by comparing the peak area of each compound observed using the original and the newly optimized CE method. The original and the optimized CEs were the same for 114 compounds; however, for 195 compounds, the signal intensity increased. Among them, the signal intensity of 125 compounds increased by 110%–150% and that of 56 substances increased by >150% but <500%. In addition, there were 14 compounds for which the signal intensity increased by a scale factor of >5 (Fig 1D). For the newly prohibited compounds, which set reference CE through direct infusion, the optimized CEs were similar to the original CEs (S1 Fig) and the signal intensity showed no differences (<110% differences). These findings confirm that the results of Skyline CE optimization do not considerably differ from the results by direct infusion. The improvements in the detectability of target compounds highlight the advantageous effect of Skyline in performing the periodical CE optimization of each compound, which is a time- and resource-intensive process, to ensure high sensitivity of the analysis.

### Quality control of intrabatch/interbatch detection quality

**Fig 2:**
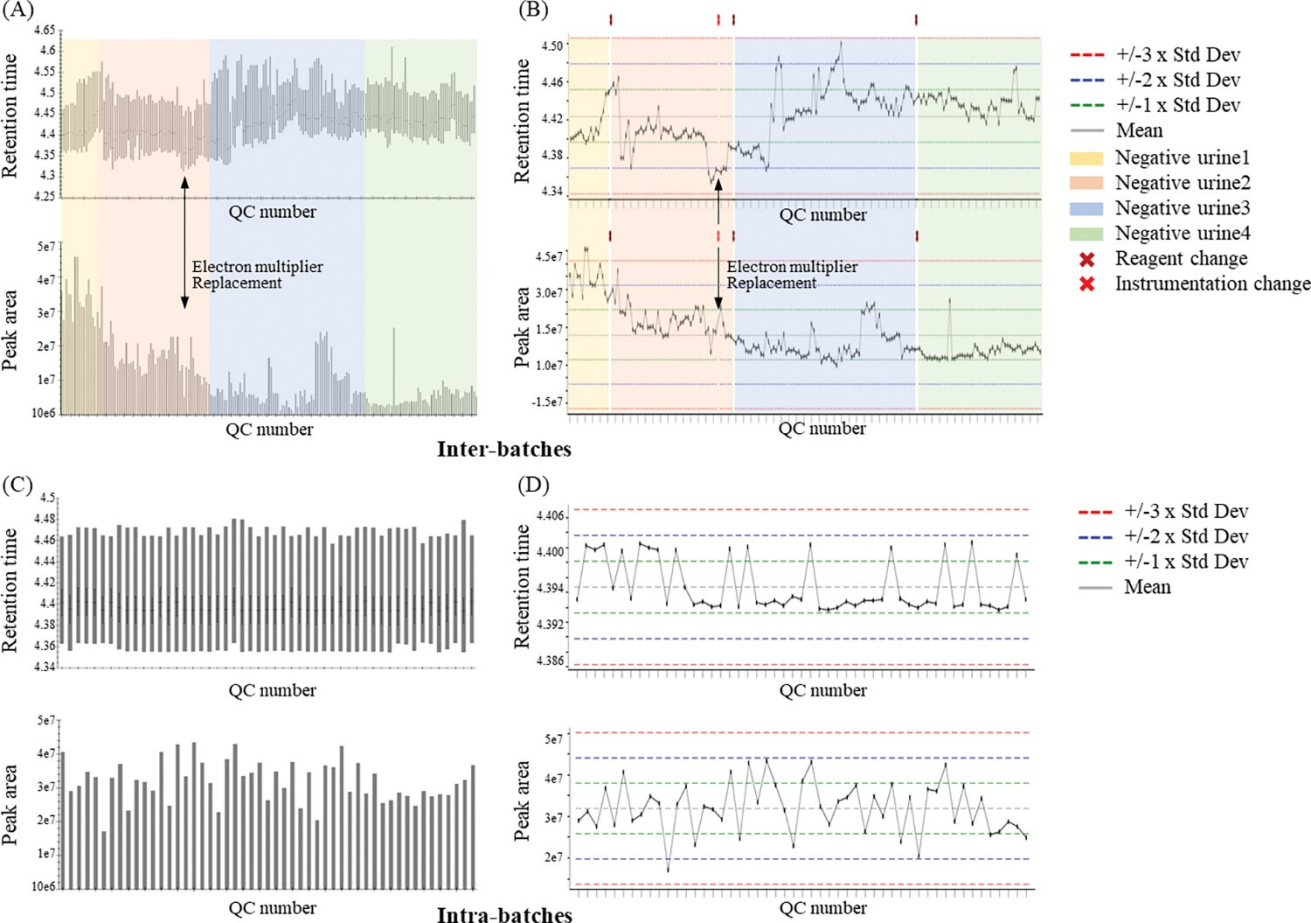
(A) and (C) Bar graph within Skyline and (B) and (D) Levey–Jennings charts within PanoramaWeb representing the standard deviation of the retention times and peak areas of internal standards in samples obtained from 2020 to 2022. The graphs show the variation of retention times and peak areas of ethyltheophylline (ISTD) in interbatches and intrabatches. The graphs indicate the detectability of the internal standard, enabling researchers to recognize the efficiency of the analysis and the status of instruments.

For longitudinal quality monitoring of sample detection, the data of 187 negative controls acquired from 2020 to 2022 were imported into Skyline. The ISTD of negative controls in whole batches and of whole samples in a single batch were subject to visualize the batch-to-batch and within-batch detection quality, respectively (Fig 2). In Skyline, the peak area or retention time of internal standard (ISTD), ethyltheophylline, is visualized in the form of a bar graph (Fig 2A and 2C), and these results can be transferred into a data repository known as PanoramaWeb (S3 Text). In PanoramaWeb, the Levey–Jennings charts providing the standard deviations of QC metrics, such as the retention time and peak area, are automatically generated (Fig 2B and 2D). According to the accumulated data, the standard deviations of QC metrics were calculated and major QC annotations, such as matrix urine changes and MS part exchange were assigned. During interbatch monitoring from April 2020 to November 2022, the matrix of the samples was changed three times and the electron multiplier was replaced in April 2021; however, the detectability was stable, showing a variation within two standard deviations. Because the Levey–Jennings charts indicate the warning limits (two standard deviations) that show trends of insufficient analytical performance and the control limits (three standard deviations), which are suggestive of significant analytical problems, the analyst could clearly understand the possible systemic error during the analysis [25, 26].

### Method validation for the newly designated prohibited compounds

Annually, the WADA prohibited list adds new substances that have potential performance-enhancing effects. Every renewal, development, or modification of the analytical method for substances listed as prohibited in sports has been a priority in the antidoping testing society. To confirm the utility of the analytical method for newly designated prohibited drugs, a delicate and robust method validation is necessary [27]. The standardized validation criteria include selectivity, reliability of detection, linearity, intraday and interday precision, sample extract stability, LOD, LOI, carryover, the matrix effect, and recovery tests. The integration of the results of all these criteria also highlights the necessity of unifying software programs that enable automated peak integration and visual inspection with multiple graph options [28]. In this study, we established an analytical method for eight substances categorized in the S6 stimulants and processed all validation procedures using Skyline[29].

The validation parameters are summarized in Table 1. Selectivity of the method was proven based on the absence of signal interference during the detection of analytes in 10 blank urine samples. The reliability of detection was verified using the MRPL based on the WADA technical document TD2019MRPL. The results acquired from all the 10 MRPL samples showed that the developed method may be considered reliable in detecting target compounds. Further, as a result of carryover, the following blanks after 400% MRPL had no detection of target materials.

**Table 1:** Method-validation summary.

Analyte	RT (min)	MRM (CE, eV)	Linearity (R^2^)	Recovery(%)	Matrix effects (%)	LOD (ng/mL)	LOI (ng/mL)	Intraday precision CV (%)	Interday precision CV (%)
S6 Stimulants (MRPL = 100 ng/mL)									
1-(-4-Fluorophenyl)-2-(methylamino)pentan-1-one	4.15	(+) 210.19 > 192.16 (13)	0.9939	94.70	96.48	1	10	1.20	18.03
		(+) 210.19 > 150.00 (18)	0.9934	94.55	95.98			1.43	17.16
		(+) 210.19 > 108.91 (23)	0.9934	94.78	95.69			1.40	18.42
2,5-Dimethoxyphenetylamine	3.46	(+) 182.17 > 165.08 (11)	0.9937	96.08	76.13	1	1	1.11	17.22
		(+) 182.17 > 150.08 (19)	0.9931	95.48	76.19			1.02	15.96
		(+) 182.17 > 135.00 (28)	0.9918	95.77	76.13			1.23	16.01
6-APB	3.77	(+) 176.16 > 159.16 (10)	0.9955	95.12	64.32	1	2	0.92	17.50
		(+) 176.16 > 131.08 (18)	0.9950	94.58	65.08			0.81	17.13
		(+) 176.16 > 91.08 (29)	0.9953	94.78	64.84			0.95	17.40
Methcathinone	2.45	(+) 164.16 > 146.00 (13)	0.9905	94.58	110.87	1	1	1.10	13.78
		(+) 164.16 > 131.08 (20)	0.9913	94.25	110.89			1.04	14.50
		(+) 164.16 > 130.08 (31)	0.9915	94.09	110.62			0.95	14.31
Methylenedioxypyrovalerone	4.34	(+) 276.23 > 205.00 (18)	0.9929	95.62	71.17	1	1	1.24	18.75
		(+) 276.23 > 175.16 (22)	0.9939	95.59	71.56			1.21	19.36
		(+) 276.23 > 126.08 (26)	0.9921	95.91	71.41			1.34	19.34
*N*-ethyl heptedrone	5.40	(+) 234.26 > 216.08 (14)	0.9931	93.18	64.47	5	10	1.10	19.20
		(+) 234.26 > 146.08 (19)	0.9928	93.10	63.73			1.15	18.63
		(+) 234.26 > 118.00 (24)	0.9932	93.30	64.63			1.13	18.93
*N*-methylphenethylamine	2.18	(+) 136.16 > 105.00 (14)	0.9947	95.35	98.79	1	1	1.18	17.05
		(+) 136.16 > 103.00 (24)	0.9945	95.39	99.00			1.19	16.55
		(+) 136.16 > 77.08 (32)	0.9944	95.11	98.73			1.13	16.18
*N*-ethylcathinone	2.82	(+) 178.18 > 160.17 (13)	0.9926	94.16	106.73	5	5	0.96	13.30
		(+) 178.18 > 132.12 (17)	0.9918	94.17	108.50			0.88	13.45
		(+) 178.18 > 131.08 (20)	0.9909	93.90	107.21			0.93	13.51

Eighteen MRPL samples separated into three groups of six samples each were used to evaluate two types of precisions. The peak-area variation of <20% within a group or within all the 18 MRPL samples indicated an intra-assay precision or an interassay precision, respectively. This result is shown in Table 1. The international criteria intended for bioanalytical methods, a CV of <20% for low analyte concentration and a CV of <15% for other concentration levels were satisfied.

The sample stability was measured with respect to the decreased rate of peak areas based on six time points: 1, 2, 3, 7, 14, and 21 days after the sample preparation In general, the detectability of compounds was maintained at >70% for 3 days. However, 7 days after sample preparation, all compounds experienced a 51.87%–66.78% reduction in detectability compared to the fresh samples.

Skyline automatically generates the bar graph of the peak area of each compound based on the RT transition. Utilizing the bar graph, the stability of each sample was easily and clearly monitored (S2 Fig). Further, the stability of analytes during 21 days are specified in Table 2. The linearity of the analytes was assessed using seven concentrations of analytes in triplicates. All data for this test are shown in Table 1. The coefficient of linearity (R^2^) for each transition ranged from 0.9905 to 0.9955.

**Table 2:** Method-validation summary; stability.

Analyte	RT (min)	MRM (CE, V)	Stability (%)					
			1 day	2nd day	3rd day	7th day	14th day	21st day
S6 Stimulants (MRPL = 100 ng/mL)								
1-(-4-Fluorophenyl)-2-(methylamino)pentan-1-one	4.15	(+) 210.19 > 192.16 (13)	100.00	92.90 ± 6.18	78.47 ± 5.51	60.18 ± 12.49	51.83 ± 11.40	45.12 ± 10.19
		(+) 210.19 > 150.00(18)	100.00	90.96 ± 9.48	77.17 ± 9.73	59.73 ± 14.44	51.65 ± 12.96	44.74 ± 11.68
		(+) 210.19 > 108.91(23)	100.00	87.27 ± 16.53	73.90 ± 15.50	56.43 ± 17.16	48.43 ± 15.25	42.40 ± 13.73
2,5-Dimethoxyphenetylamine	3.46	(+) 182.17 > 165.08(11)	100.00	90.01 ± 6.94	75.68 ± 9.00	58.51 ± 14.45	50.34 ± 12.85	43.13 ± 11.70
		(+) 182.17 > 150.08(19)	100.00	88.42 ± 13.89	74.68 ± 13.13	58.21 ± 16.11	50.63 ± 14.48	44.14 ± 13.22
		(+) 182.17 > 135.00(28)	100.00	85.93 ± 21.54	72.46 ± 18.58	55.73 ± 18.50	48.52 ± 16.62	42.75 ± 15.19
6-APB	3.77	(+) 176.16 > 159.16(10)	100.00	85.55 ± 16.67	72.56 ± 16.82	54.76 ± 17.81	45.69 ± 15.99	39.34 ± 14.64
		(+) 176.16 > 131.08(18)	100.00	86.13 ± 17.01	72.59 ± 16.41	53.76 ± 17.33	44.92 ± 15.74	38.75 ± 14.54
		(+) 176.16 > 91.08(29)	100.00	82.89 ± 25.53	70.02 ± 22.03	51.87 ± 19.66	43.34 ± 17.05	37.67 ± 15.54
Methcathinone	2.45	(+) 164.16 > 146.00(13)	100.00	101.36 ± 31.23	86.42 ± 20.88	66.78 ± 15.00	57.76 ± 12.90	49.15 ± 11.69
		(+) 164.16 > 131.08(20)	100.00	96.73 ± 18.40	82.05 ± 10.80	62.30 ± 10.71	53.79 ± 9.58	47.44 ± 9.06
		(+) 164.16 > 130.08(31)	100.00	91.50 ± 7.17	77.51 ± 4.52	58.57 ± 10.84	50.80 ± 9.82	45.47 ± 9.48
Methylenedioxypyrovalerone	4.34	(+) 276.23 > 205.00(18)	100.00	89.13 ± 9.15	75.71 ± 10.33	58.03 ± 14.25	49.51 ± 12.11	42.97 ± 11.46
		(+) 276.23 > 175.16(22)	100.00	89.00 ± 7.88	75.24 ± 10.01	57.48 ± 13.96	48.89 ± 12.25	43.54 ± 11.54
		(+) 276.23 > 126.08(26)	100.00	89.99 ± 6.09	76.79 ± 9.61	59.61 ± 14.03	51.21 ± 12.32	44.57 ± 11.29
*N*-ethyl heptedrone	5.40	(+) 234.26 > 216.08(14)	100.00	100.20 ± 26.81	83.96 ± 16.98	63.63 ± 13.83	54.70 ± 12.42	45.42 ± 10.94
		(+) 234.26 > 146.08(19)	100.00	92.10 ± 4.57	78.58 ± 7.70	61.15 ± 13.41	53.04 ± 12.25	45.76 ± 10.98
		(+) 234.26 > 118.00(24)	100.00	88.69 ± 12.65	75.51 ± 13.16	58.03 ± 15.73	50.44 ± 14.44	43.93 ± 12.90
*N*-methylphenethylamine	2.18	(+) 136.16 > 105.00(14)	100.00	97.33 ± 18.91	81.97 ± 11.87	61.98 ± 11.98	53.37 ± 10.74	46.50 ± 9.89
		(+) 136.16 > 103.00(24)	100.00	85.40 ± 22.01	72.77 ± 19.31	55.31 ± 18.45	47.71 ± 16.16	42.32 ± 14.80
		(+) 136.16 > 77.08(32)	100.00	87.37 ± 16.51	74.69 ± 15.54	57.36 ± 16.75	49.51 ± 14.58	44.05 ± 13.49
*N*-ethylcathinone	2.82	(+) 178.18 > 160.17(13)	100.00	89.12 ± 8.28	76.14 ± 9.99	59.29 ± 13.75	50.85 ± 11.90	45.77 ± 11.35
		(+) 178.18 > 132.12(17)	100.00	88.27 ± 12.09	75.09 ± 11.95	57.71 ± 14.42	49.61 ± 12.79	44.33 ± 11.85
		(+) 178.18 > 131.08(20)	100.00	86.64 ± 16.34	74.26 ± 15.76	57.67 ± 16.84	49.67 ± 14.79	43.74 ± 13.38

The LODs of all compounds were ≤5% of the MRPL. Most analytes showed suitable detection around the 1 ng/mL concentration. In addition, based on the relative abundances of three transitions for each compound, we could measure the LOI of each compound. According to the relative abundance of each transition based on the major diagnostic ion, concentrations that met the categorized criteria were chosen as the LOI. The results are shown in Table 1.

The rate of recovery of each compound ranged from 93.10% to 96.08%. The existence of either an underestimation or an overestimation of the concentration of analytes was evaluated by the matrix effect. Less than 20% of the absolute value of the matrix effect is considered as the soft matrix effect; 1-(4-fluorophenyl)-2-(methylamino) pentane-1-one, methcathinone, *N*-methylphenethyl amine, and *N*-ethylcathinone. The medium matrix effect, which is >20% and <50% of the absolute value, was observed in case of 2,5-dimethoxyphenetylamine, 6-APB, methylenedioxypyrovalerone, and *N*-ethyl heptedrone [30]. Additional techniques that mitigated the effects of the matrix, such as the dilution of the sample before the preparation, were partially needed [31].

### ITP screening

**Fig 3:**
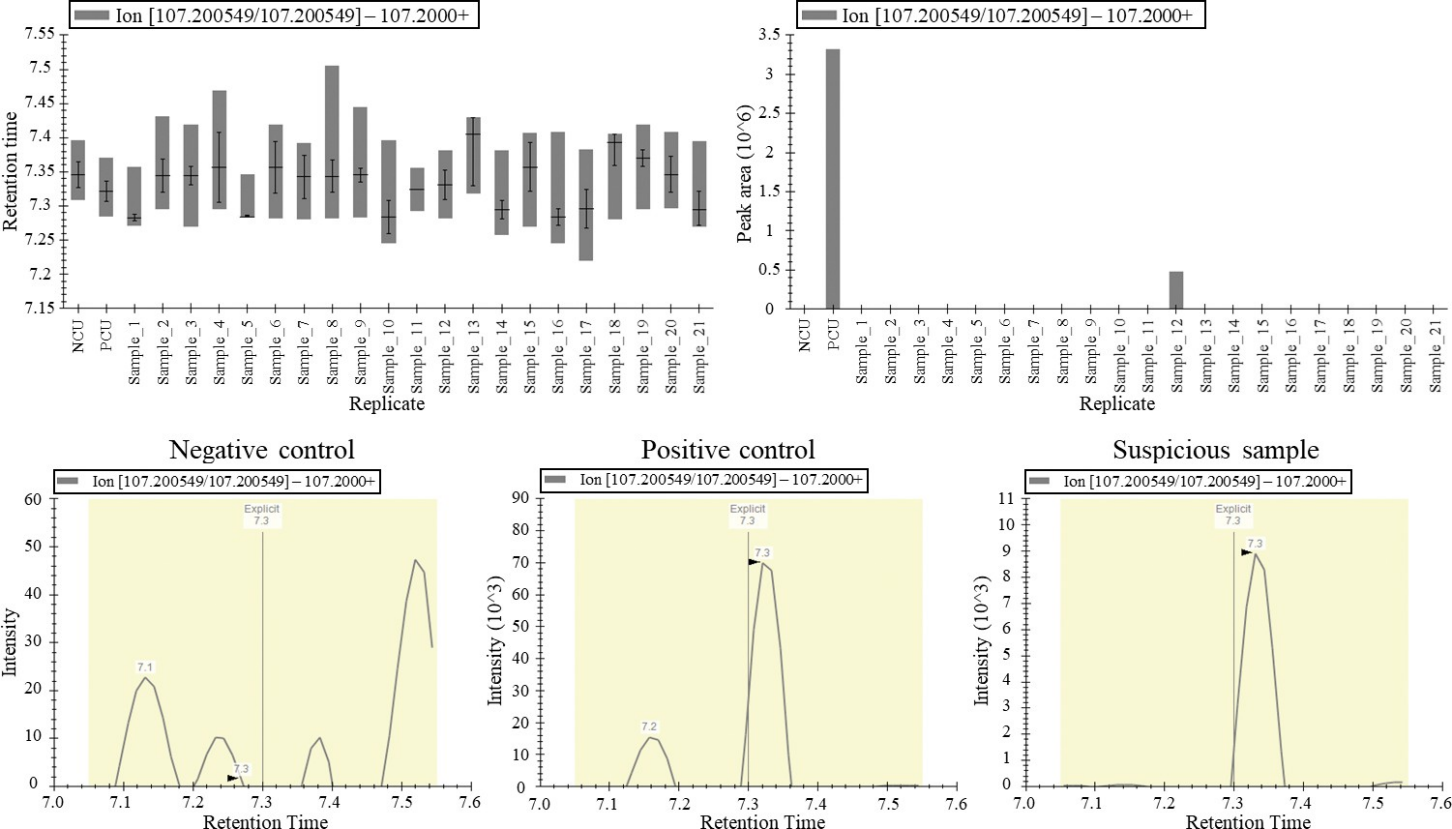
ITP screening using Skyline. The upper left corner of the figure shows the distribution of the retention time. The upper right corner of the figure shows the bar graph of the normalized peak area in a batch. The chromatogram for canrenone on the negative control, positive control, and suspicious samples are shown respectively at the second row. The detected compound was canrenone, analyzed based on the transition pair of 341.2 → 107.2.

To confirm the applicability of Skyline for screening prohibited substances, a trial initial test procedure (ITP) batch comprising 21 urine samples was used. Among them, 19 samples were negative and the remaining two were artificially spiked with stanozolol, anastrozole, and canrenone. Information about samples, such as the existence of positive samples and the composition of prohibited compounds, was confidential. After the LC-MS/MS analysis, raw data were directly imported into Skyline, which had a predesigned layout showing the distribution of the retention time, bar graph of peak areas, and extracted ion chromatogram of predefined ion pairs of target drugs (S1 Text). Skyline provides numerous options for users to generate customized layouts. After importing raw data into Skyline, the retention times (Fig 3, upper left) and peak areas (Fig 3, upper right) of 247 prohibited substances in each sample were automatically shown in the form of bar graphs. The analyst could identify suspicious samples by comparing the bar graphs and chromatograms of negative controls, positive controls, and suspicious samples (Fig 3). Samples suspected to contain canrenone, stanozolol, and anastrozole were intuitively screened based on the bar graph of the peak area and double-checked using the chromatogram. Sample number 12 showed an abnormal level of canrenone compared with the others.

To verify the advantages of Skyline software in data screening over commercial softwares, a comparison analysis was conducted between Skyline and TraceFinder, commercially available software. The evaluation focused on the time required for reviewing and the number of suspicious samples identified. The study involved reviewers with varying levels of experience, including early trainees (with two months to one year of training) and advanced trainees (with over two years of experience). Results showed considerably longer reviewing time for TraceFinder than Skyline (Tables 3 and S1 Table). Specifically, the reviewing time using TraceFinder was 3.07 times longer for early trainees (*p*-value 0.0003 by the independent t-test), and 1.71 times longer for advanced trainees (*p*-value 0.0041 by the independent t-test) than using Skyline. Neither software platform produced any false negatives, but TraceFinder identified more suspicious compounds than Skyline. After ITP screening, the selected suspicious samples undergo a confirmation procedure (CP) test, a more sophisticated method using targeted methods that utilize more than three transitions of suspicious substances and calculate the relative abundance among the transition pairs to determine their actual positive status regarding doping. Therefore, the primary purpose of the ITP is to improve the efficiency of subsequent CP by selecting suspicious samples and minimizing the selection of false positives. Although the Mann–Whitney U test showed no statistical difference between the two software platforms, the number of suspicious compounds requiring reanalysis to confirm positive samples was 1.95 and 1.56 times higher when using TraceFinder than those when using Skyline. The statistical analysis is limited by the small number of participants. Nonetheless, the results indicated that the ability to screen suspect samples did not differ considerably between the two softwares. However, the time spent on reviewing was substantially reduced in both trainee groups when using Skyline. From this perspective, the benefits of utilizing Skyline for ITP screening have been confirmed.

**Table 3:** Comparison of doping screening efficiency between Skyline and commercially available TraceFinder.

	TraceFinder		Skyline			*p*-value (TraceFinder vs Skyline)		Fold change (TraceFinder / Skyline)	
	Early	Advanced	Early	Advanced		Early	Advanced	Early	Advanced
	trainee (n = 4)	trainee (n = 2)	trainee (n = 4)	trainee (n = 2)		trainee (n = 4)	trainee (n = 2)	trainee (n = 4)	trainee (n = 2)
Cost (software maintenance)	$6,878		Open source			-	-	-	-
Time (min)	54.7 ± 8.6	26.5 ± 0.7	18.5 ± 3.8		15.5 ± 0.7	0.0003	0.0041	3.07 ± 0.9	1.71 ± 0.1
Suspicious compound	25.7 ± 10.4	12.5 ± 4.9	13.5 ± 5.9		8 ± 0.0	0.0877	0.1213	1.95 ± 0.3	1.56 ± 0.6

The *p*-value was calculated by the independent t-test or Mann–Whitney U test.

### Quantitative analysis

**Fig 4:**
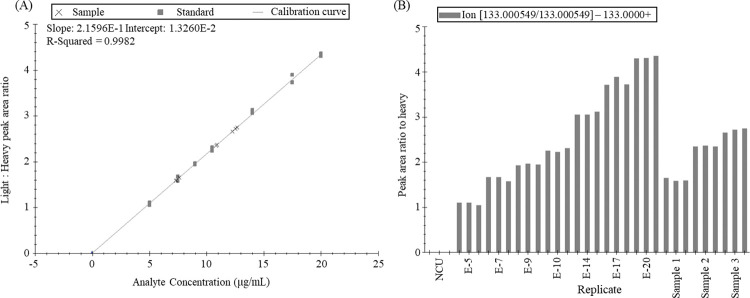
Quantitative analysis conducted using Skyline. (A) Calibration curve of seven calibrators and (B) bar graph of the peak area of all imported samples were automatically generated. The ephedrine concentration was normalized with d3-ephedrine by dividing the ephedrine peak area with that of stable isotope. Seven calibrators spiked with 5, 7, 9, 10, 14, 17, and 20 μg/mL of ephedrine were used to generate the calibration curves. The nominal concentrations of each sample were 7.4, 11.1, and 12.3 μg/mL. The analysis of all samples exhibited satisfactory precision and accuracy. For Sample 1, CV% = 2.33 and accuracy = 99.99%. For Sample 2, CV% = 0.44 and accuracy = 97.88%. For Sample 3, CV% = 1.71 and accuracy = 101.47%.

Some antidoping drugs require quantitative analysis. Quantitative protein analysis has been performed using Skyline in many previous studies. In particular, Seo et al. analyzed IGF-1 protein levels in the blood to perform a doping analysis and derived quantitative analysis results through Skyline [32]. However, there have been few studies that quantified substances such as small molecules [33]. In this study, we used Skyline for obtaining the quantitative analysis results for ephedrine, one of the prohibited substances. In the molecule setting of Skyline, the linear regression fit, the ratio to heavy normalization method, and units of calibration curves were designated (S2 Text). After importing the raw data of standard samples analyzed at seven concentration points with triplicate replicates, a blank, and three unknown samples, the nominal concentrations of the calibrators and sample information such as blank, standard, and unknown were assigned in the document grid: replicates. The calibration curve was plotted, and the coefficients of determination (R^2^) of the curve and the concentration of each unknown sample were calculated using the calibration curve. (Fig 4) The standard curve based on the peak area and the seven concentration points of the standard exhibited a strong correlation (R^2^ = 0.9982). As per the reports of document grid, the user can customize the report format and export the quantitative results. The precision and accuracy of samples were calculated by reporting the sample concentration, and all samples exhibited ≤2.338 CV% and >97.88% accuracy.

## Conclusions

With steady increase in the development of new abused drugs, the number of prohibited molecules in sports drug testing and the responsibility of the doping control laboratories for upgrading an analytical methods for detecting them have increased [10]. To satisfy the requirements of high throughput for the increasing number of prohibited chemicals in sports drug testing, performing the arduous screening process with the lowest possible workload is one of the critical solutions to ensure high sensitivity and accuracy of the analysis. In this study, we proposed the use of Skyline as an easy-to-implement approach for creating a systemic process for doping control including method optimization, sample screening, longitudinal quality monitoring, and method validation. However, there is a limitation to the use of Skyline as a major tool for reporting doping result because it does not support image files to be exported based on the layout and can only export data in the csv and tsv formats. Every figure including the chromatogram can be saved independently in the emf, png, gif, jpg, tif, and bmp formats. However, according to the WADA technical document TD2022LDOC, the documents should be reported with original file or data to prohibit their modifications. Therefore, the data that cannot eliminate the possibility of being modified, such as extracting each figure separately by the analyzer, would not be accepted as the official reporting documents. Nevertheless, the application of Skyline for doping control enables the user to optimize CEs, visually review the combined results, and quantify the analytes of interest. Further, Skyline allows experts to build a vendor-independent platform within the doping society by sharing their results. With increasing need for robust and easily-accessible tools for data processing, Skyline will become an efficient tool in the doping society.

Additionally, not only tracing the variation of analytical errors over time but also the longitudinal profiling of biological variables has been imported into doping control [34]. For athlete biological passport, differences in some of the hematologic or urinary variables over time has been monitored [35–37]. If considerable deviations were found within the athlete biological passport variables, the athlete would have to undergo additional tests to check the use of prohibited substances. This system has brought advantages to the antidoping society by providing more efficient, targeted testing and allowing effective restriction to the athlete based on the obtained information [38]. With the convenience of monitoring the longitudinal data using Skyline and Panorama, the establishment of each athlete’s biological passport would be possible.

## Supporting information

S1 Fig Number of transitions showing collision energy (CE) changes after CE optimization using Skyline. The default CE was determined through direct infusion. As the transitions already had optimized CEs through direct infusion, the difference between the original and optimized CE was not statistically significant. This result confirms that CE optimization by Skyline validated the CE settings for those transitions.
(TIF)

S2 Fig Bar graph depicting the decrease in peak area for samples after 1, 2, 3, 7, 14, and 21 days of sample preparation in the stability test. (TIF)

S3 Fig (TIF)

S1 Table Raw data comparing the efficiency of doping screening between skyline and the commercially available software TraceFinder. (DOCX)

S1 Text Workflow for the Skyline adaption into doping data screening. (DOCX)

S2 Text Workflow for the quantitative analysis using Skyline. (DOCX)

S3 Text Workflow for the longitudinal detection–quality-control monitoring using panorama web and Skyline. (DOCX)

## Abbreviations

LC-MS/MS: liquid chromatography based tandem mass spectroscopy
SRM: selected reaction monitoring
DDA: data-dependent acquisition
WADA: World Anti-Doping Agency
ISTD: Internal standard
WCX: weak cation exchange
LOD: limit of detection
LOI: limit of identification
MRPL: minimum required performance level
CV: coefficient of variation
