# Supplementary figures and images for: Application of Skyline software for detecting prohibited substances in doping control analysis
Source: PLoS One. 2023 Dec 5;18(12):e0295065. doi: 10.1371/journal.pone.0295065 (PMC10697575; doi:10.1371/journal.pone.0295065)

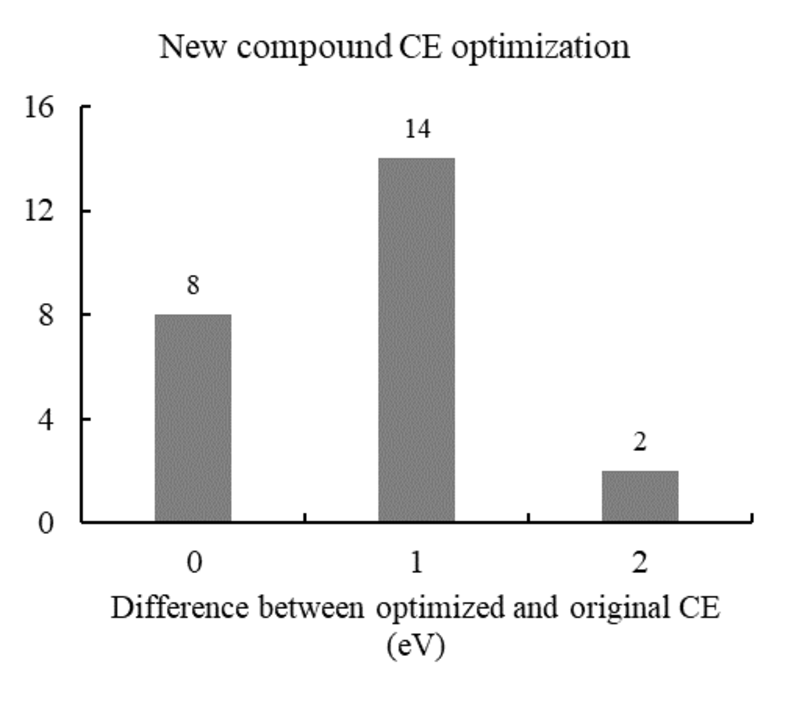

Supplement: S1 Fig — The default CE was determined through direct infusion. As the transitions already had optimized CEs through direct infusion, the difference between the original and optimized CE was not statistically significant. This result confirms that CE optimization by Skyline validated the CE settings for those transitions. (TIF) [file pone.0295065.s001.tif]

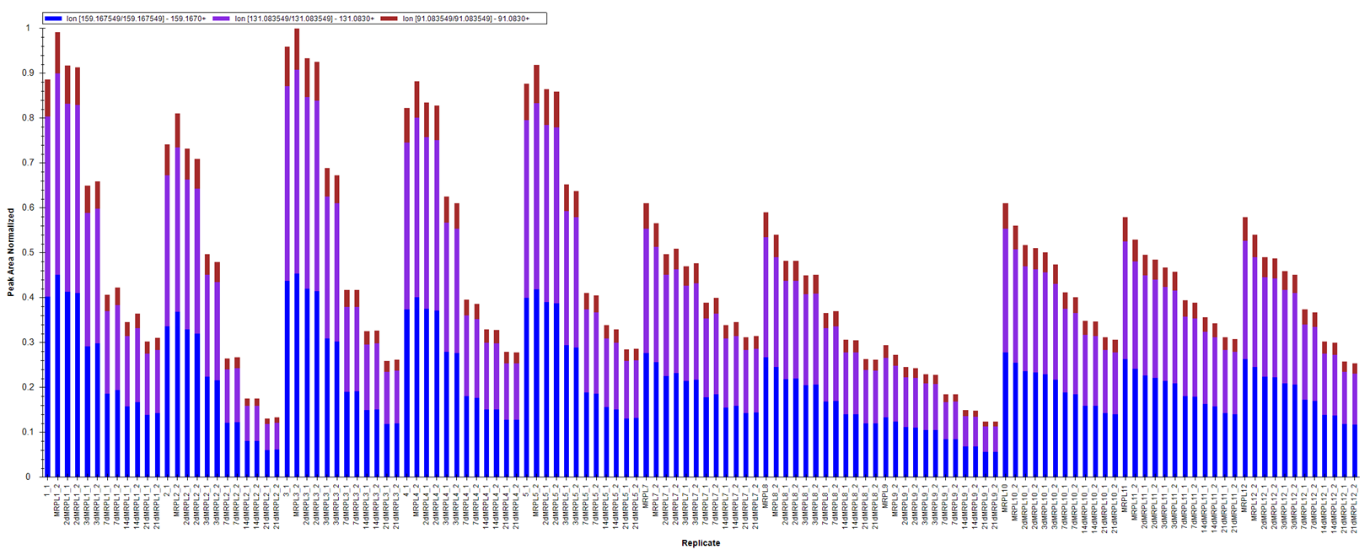

Supplement: S2 Fig — (TIF) [file pone.0295065.s002.tif]

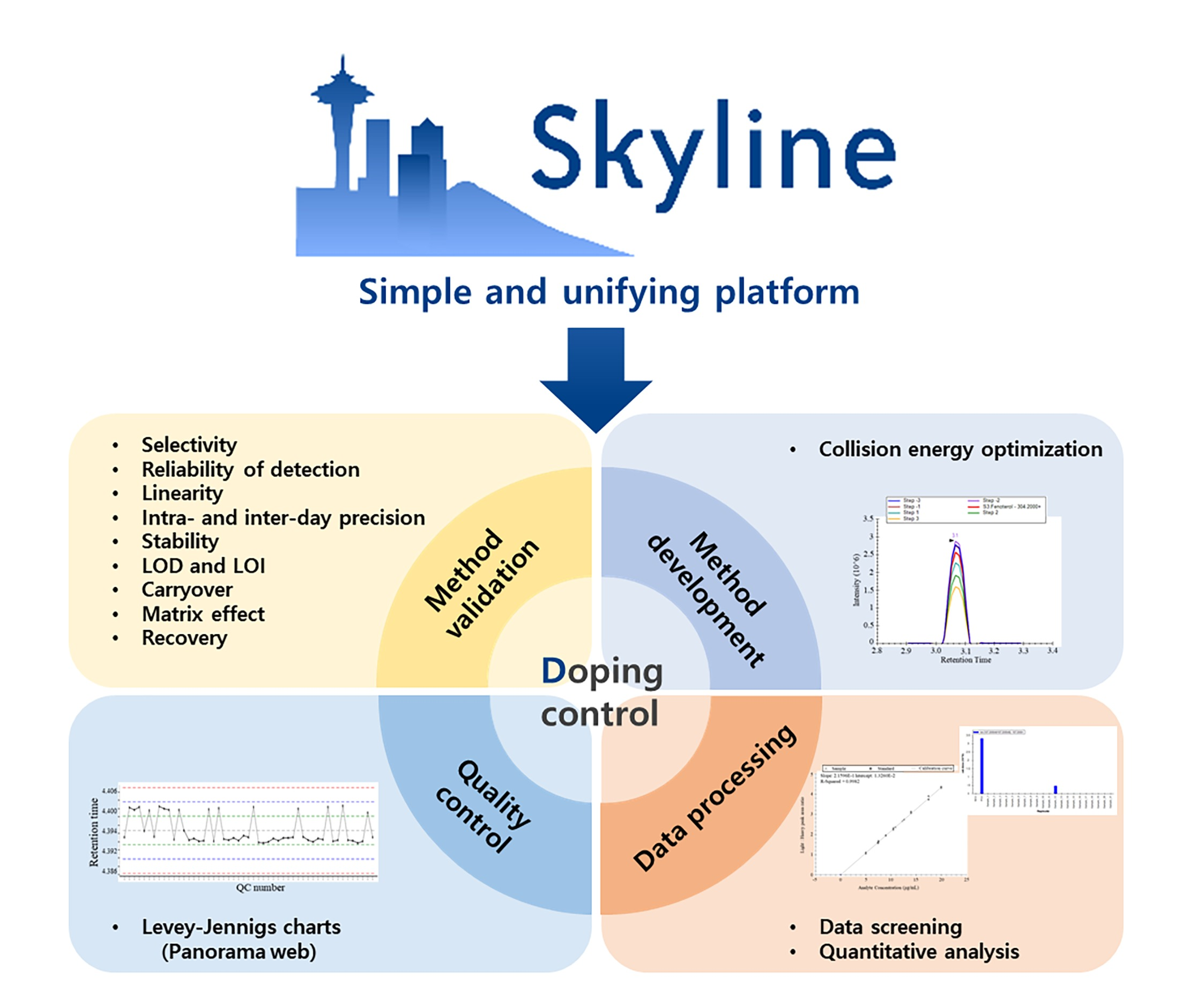

Supplement: S3 Fig — (TIF) [file pone.0295065.s003.tif]
