# Supplementary material for: Application of Skyline software for detecting prohibited substances in doping control analysis
Source: PLoS One. 2023 Dec 5;18(12):e0295065. doi: 10.1371/journal.pone.0295065 (PMC10697575; doi:10.1371/journal.pone.0295065)
Supplement: S1 Table — (DOCX) [file pone.0295065.s004.docx]

**S1 Table. Raw data comparing the efficiency of doping screening between Skyline and the commercially available software TraceFinder**

|  |  | Time (min) | | | Suspicious compounds | | |
| --- | --- | --- | --- | --- | --- | --- | --- |
|  | Software | TraceFinder (TF) | Skyline | Fold change (TF/Skyline) | TraceFinder | Skyline | Fold change (TF/Skyline) |
| Early trainee | Tester 1 | 66 | 15 | 4.40 | 19 | 8 | 2.37 |
|  | Tester 2 | 53 | 24 | 2.20 | 41 | 22 | 1.86 |
|  | Tester 3 | 55 | 18 | 3.05 | 19 | 12 | 1.58 |
|  | Tester 4 | 45 | 17 | 2.64 | 24 | 12 | 2.00 |
| Advanced trainee | Tester 5 | 26 | 16 | 1.62 | 9 | 8 | 1.12 |
|  | Tester 6 | 27 | 15 | 1.80 | 16 | 8 | 2.00 |
