# Supplementary material for: Application of Skyline software for detecting prohibited substances in doping control analysis
Source: PLoS One. 2023 Dec 5;18(12):e0295065. doi: 10.1371/journal.pone.0295065 (PMC10697575; doi:10.1371/journal.pone.0295065)

**S1 Text. Workflow for the Skyline adaptation into doping data screening**
1. Create a table containing information of prohibited substances as a csv file.
Specify positive polarity as 1 and negative polarity as −1.

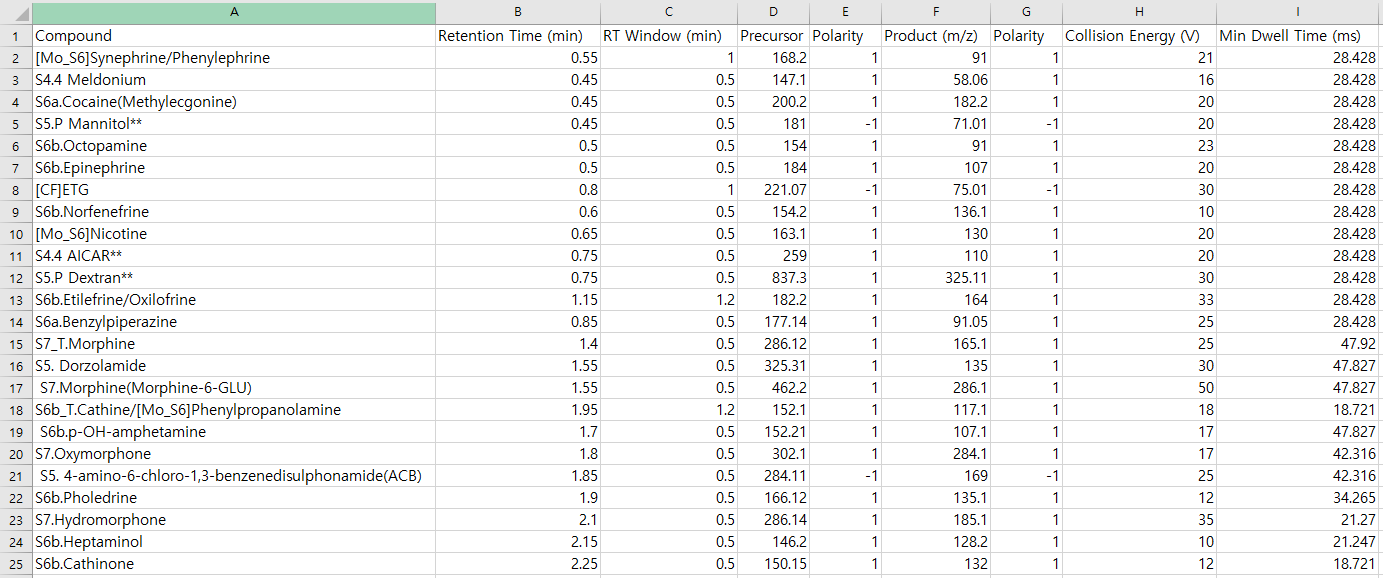

2. Open Skyline and import the csv files, specify the second row as the column information, click “Check for Errors” and “OK”.

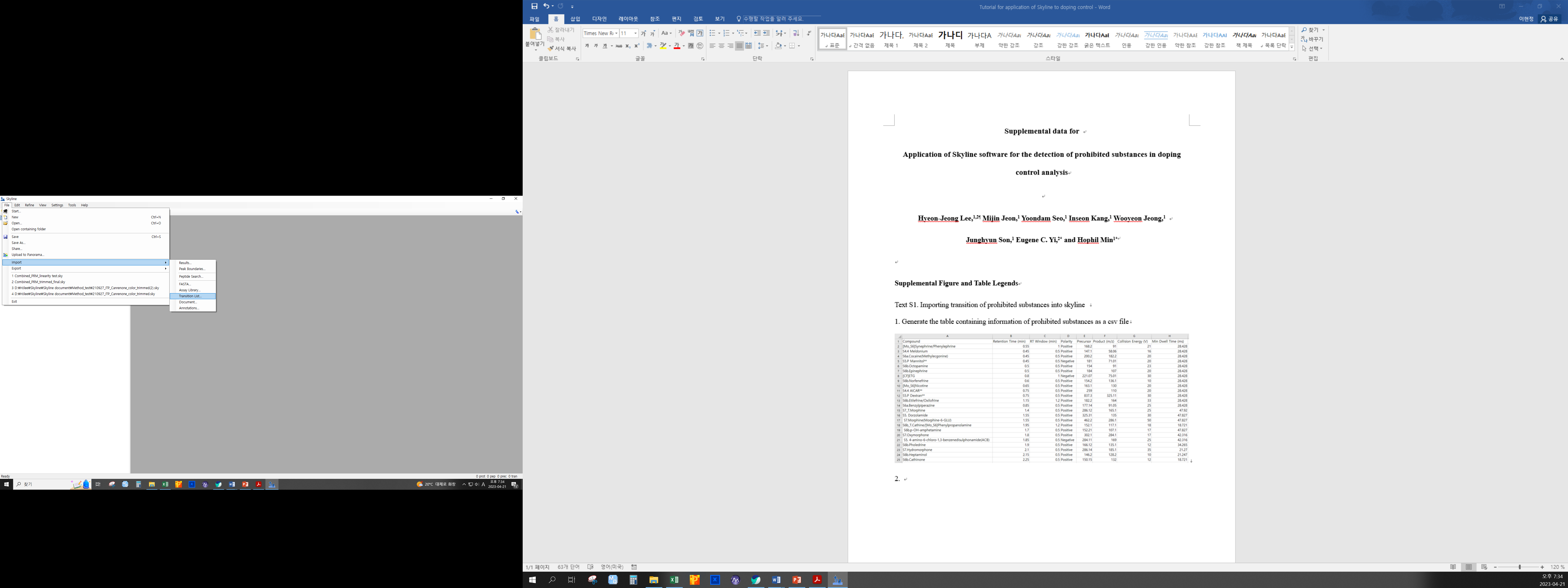


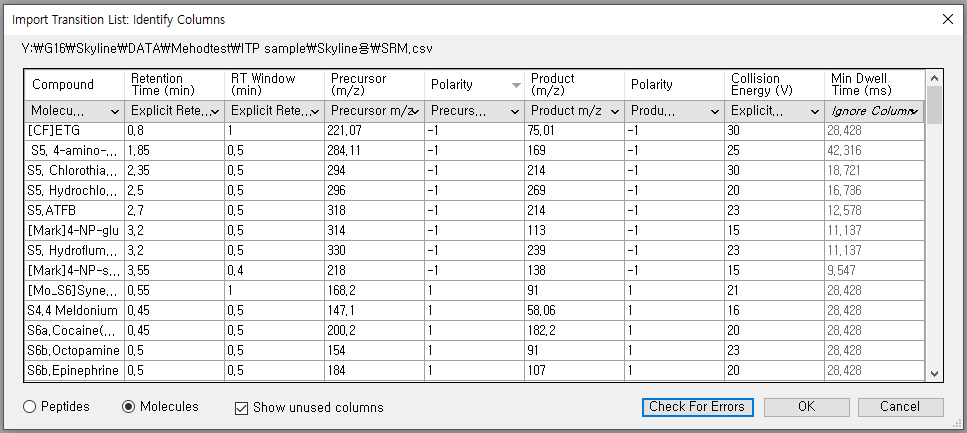

3. For SRM raw data import, match the values as shown in the following picture (Settings → Transition settings → Full-Scan).

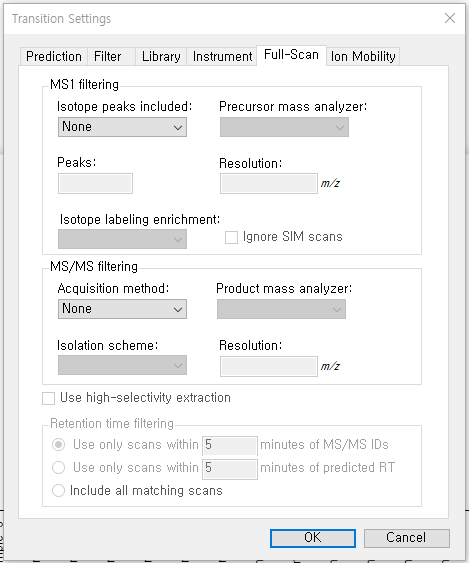


4. Import the SRM raw data into Skyline.

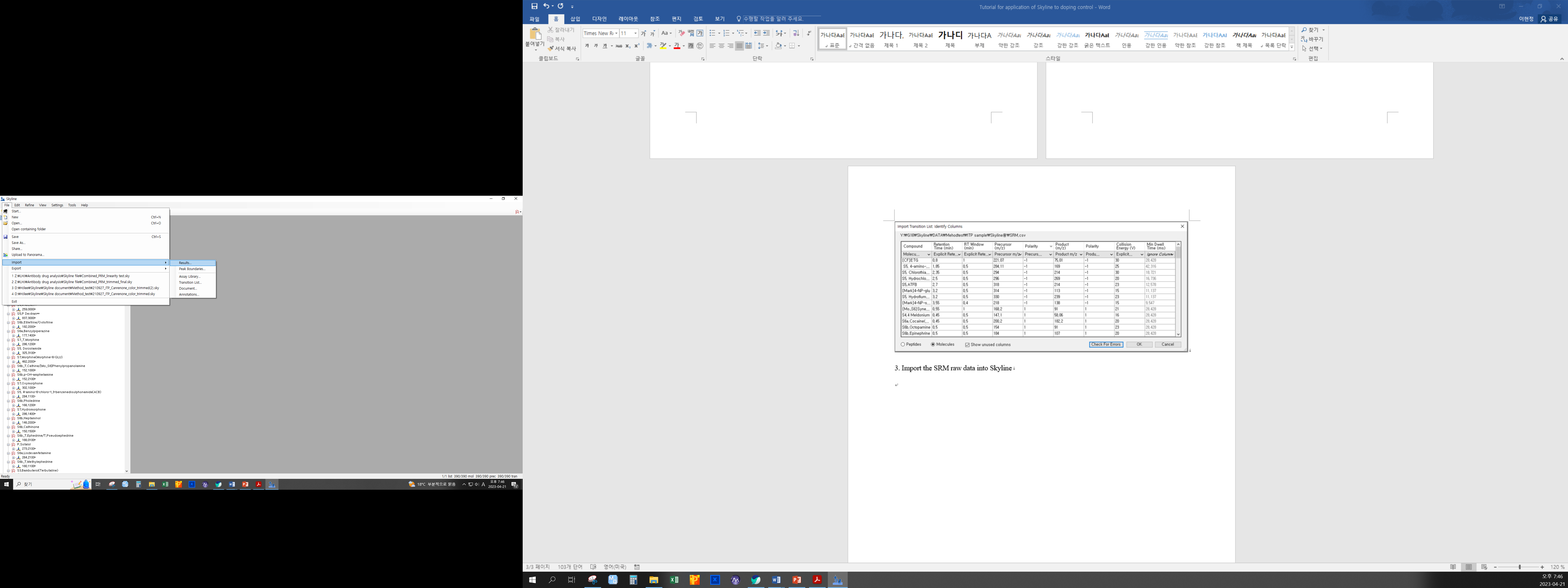

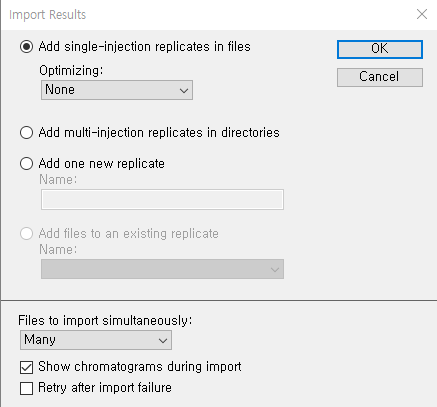

5. Screen the retention time, peak area, and extracted chromatogram.

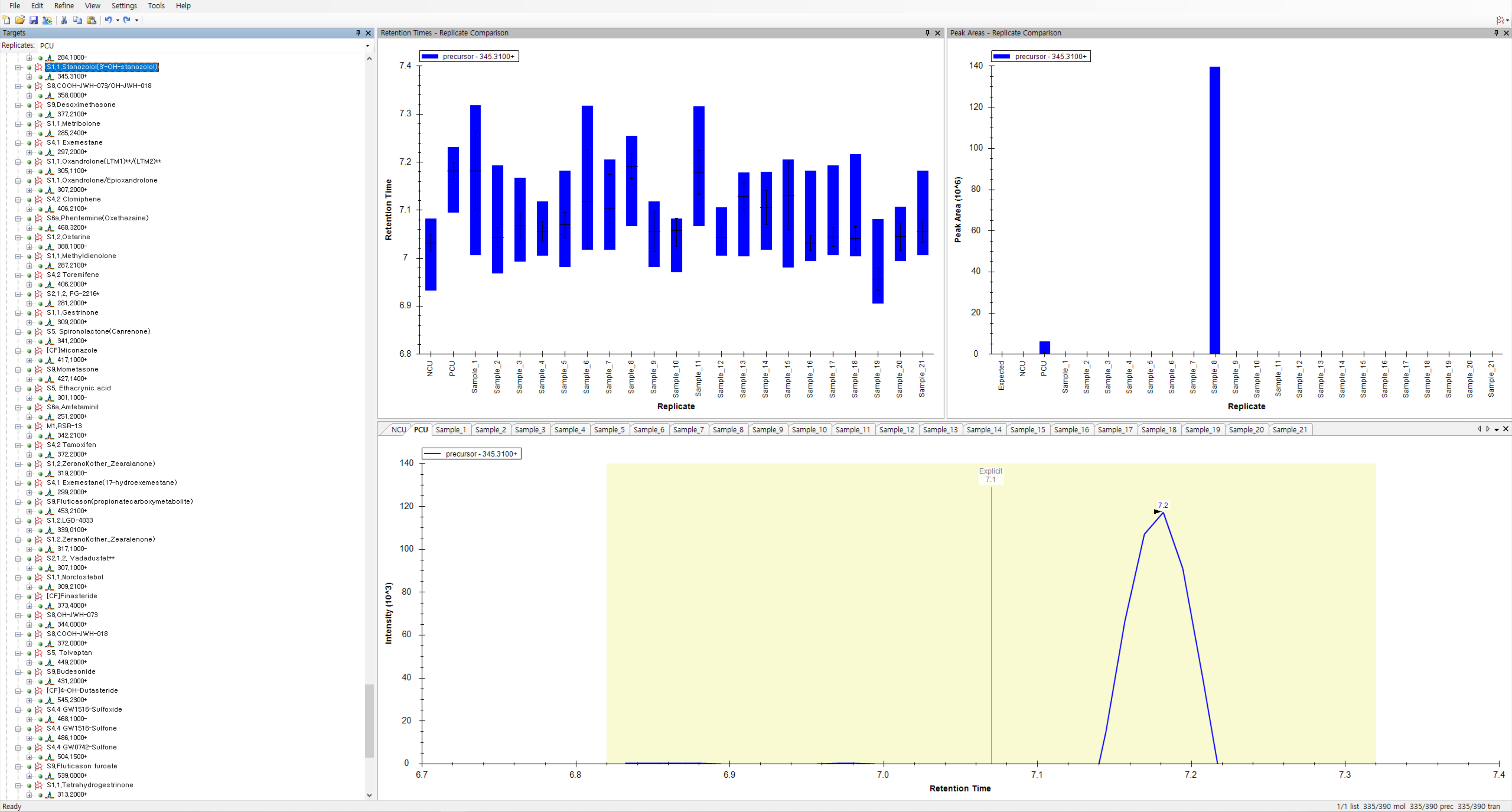

Supplement: S1 Text — (DOCX) [file pone.0295065.s005.docx]
