# Supplementary material for: Application of Skyline software for detecting prohibited substances in doping control analysis
Source: PLoS One. 2023 Dec 5;18(12):e0295065. doi: 10.1371/journal.pone.0295065 (PMC10697575; doi:10.1371/journal.pone.0295065)

**S2 Text. Workflow for the quantitative analysis using Skyline**
1. Insert transition for the target compound before data importing (refer S1 Text. Steps 1 and 2)

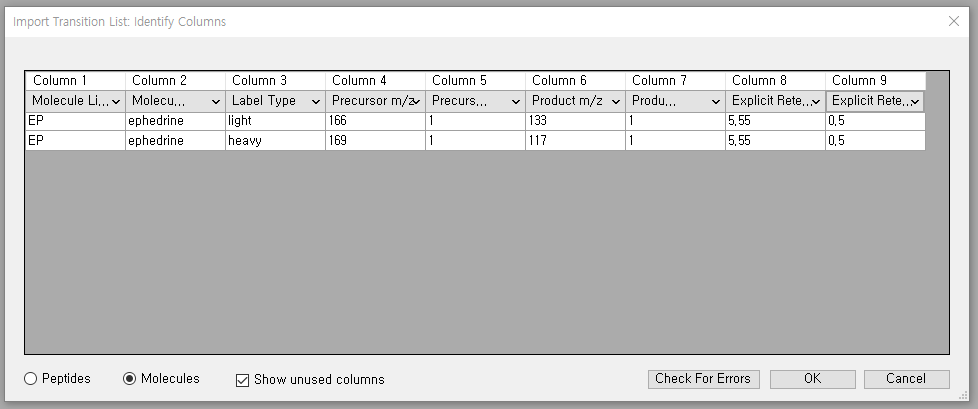


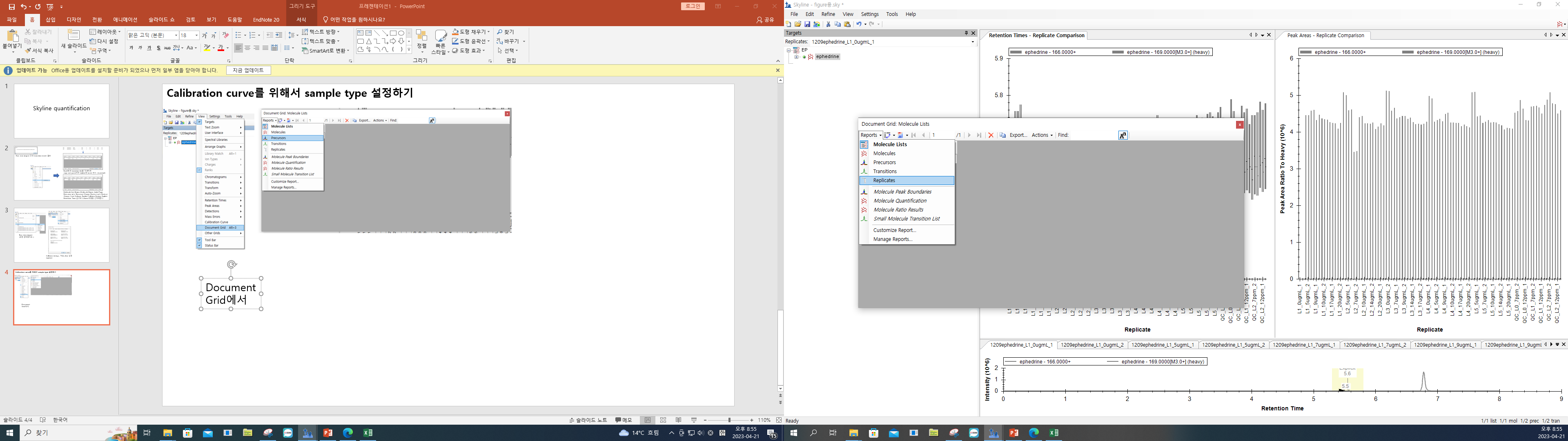

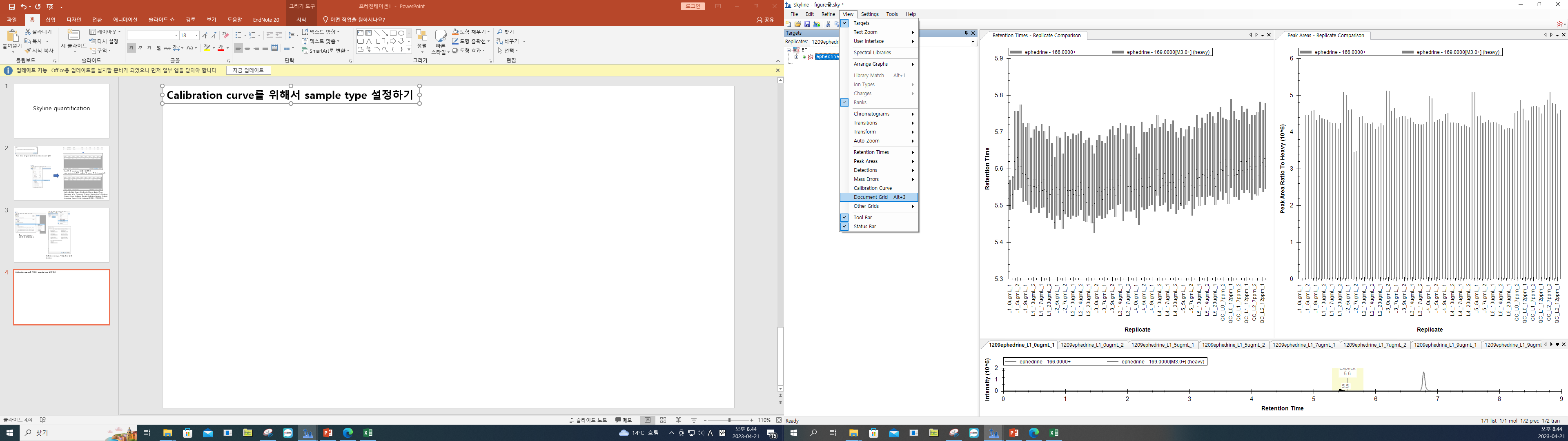
2. Import the raw data (refer to S1 Text, Step 4)

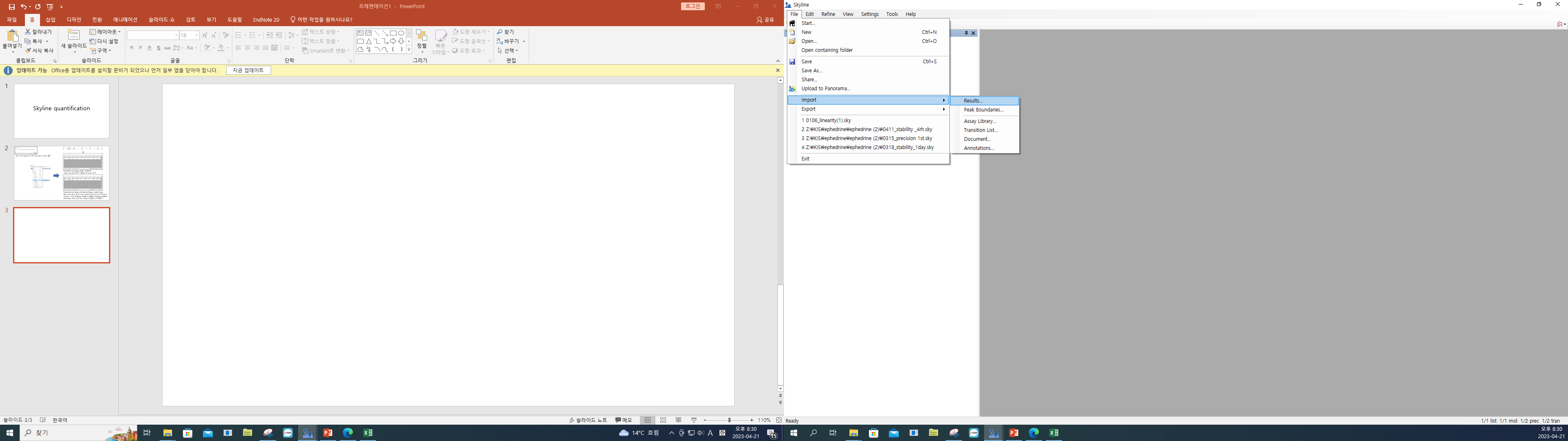

3. Within Document grid calibration curve, specify the sample type as Blank, Standard, or Unknown (for the sample) and the concentration of the calibrator for the calibration in Analyte concentration. (View → Document grid → Reports →Replicates).


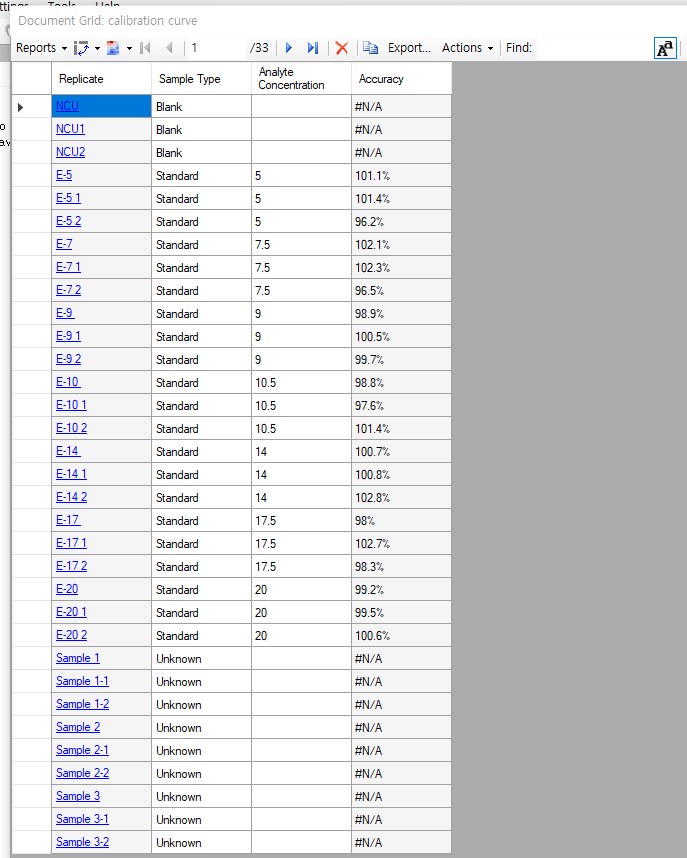


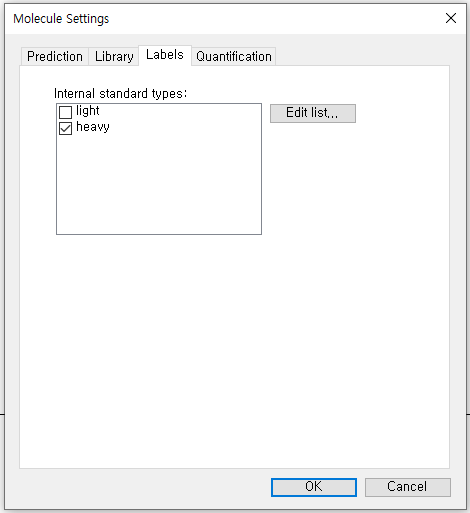

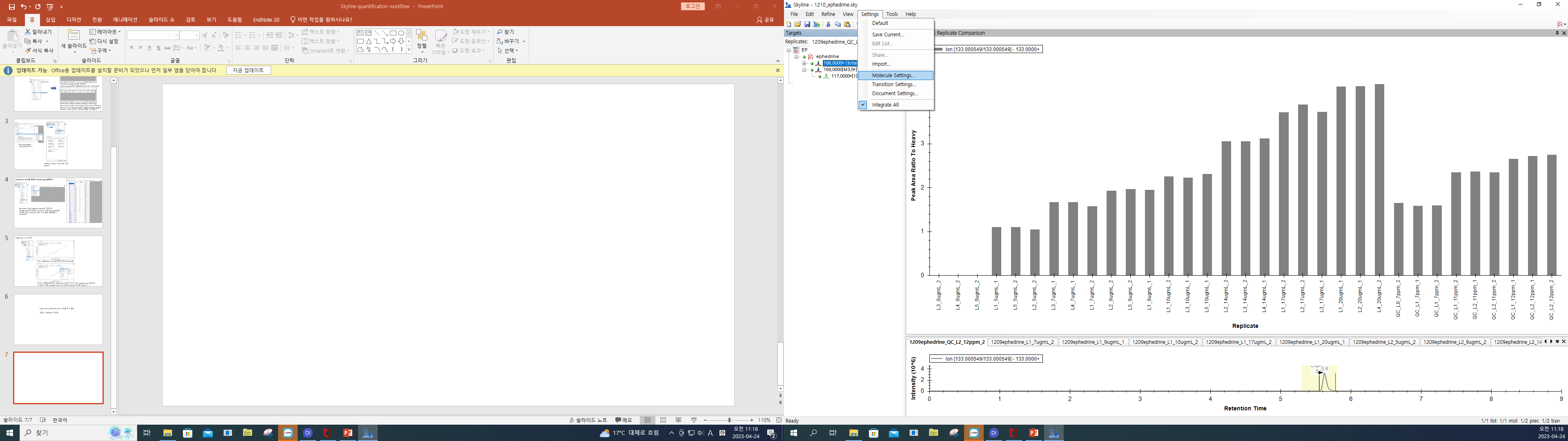
4. To quantify concentration based on the isotopic ratio, the molecule settings should be modified. (Settings → Molecule Settings → Labels → within Internal standard types: check Heavy → OK). Additionaly, (Molecule settings → Quantification → Normalization method:Ratio to Heavy).


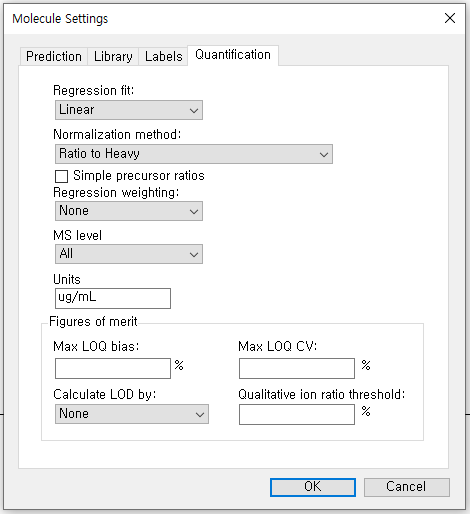


5. Create a calibration curve (View → Calibration curve). To specify the sample type to be displayed in the calibration curve (right click on the calibration curve → show sample types → select the sample type to display).

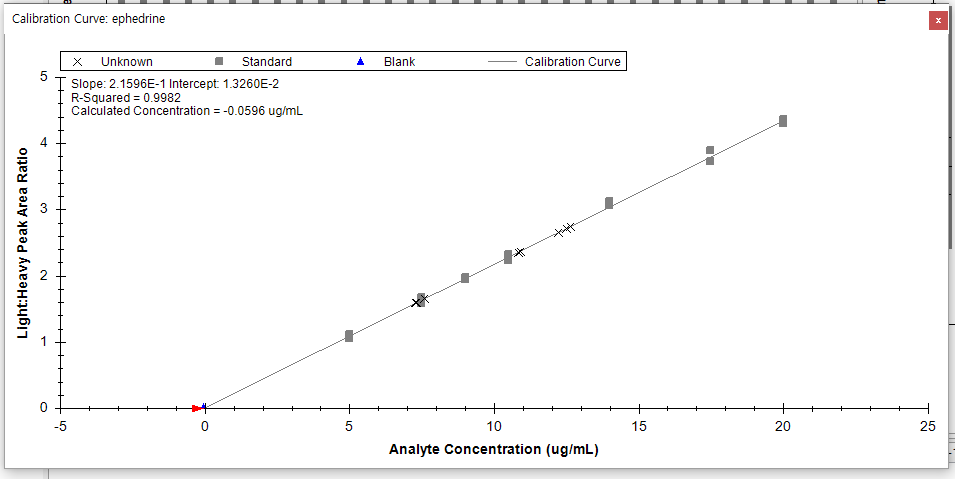


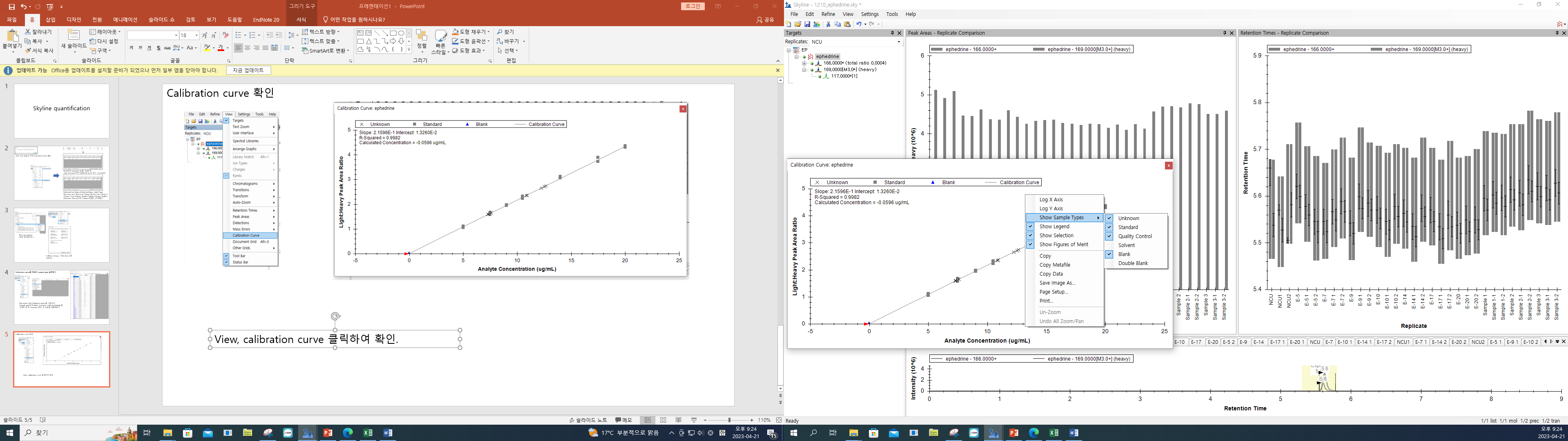

Supplement: S2 Text — (DOCX) [file pone.0295065.s006.docx]
