# Supplementary material for: Application of Skyline software for detecting prohibited substances in doping control analysis
Source: PLoS One. 2023 Dec 5;18(12):e0295065. doi: 10.1371/journal.pone.0295065 (PMC10697575; doi:10.1371/journal.pone.0295065)

**S3 Text. Workflow for the longitudinal detection–quality-control monitoring using PanoramaWeb and Skyline**

1. Sign up for an account on PanoramaWeb, and create the folder and subfolder that will act as the repository for longitudinal data.

(Projects in the upper right corner → Expend Group name folder that you assigned when you made an account → Click the folder → Click the new folder icon → Enter subfolder name → Select Panorama option for Folder type → Next → Select Inherit From Parent Folder → Next → Select Experimental data → Finish)

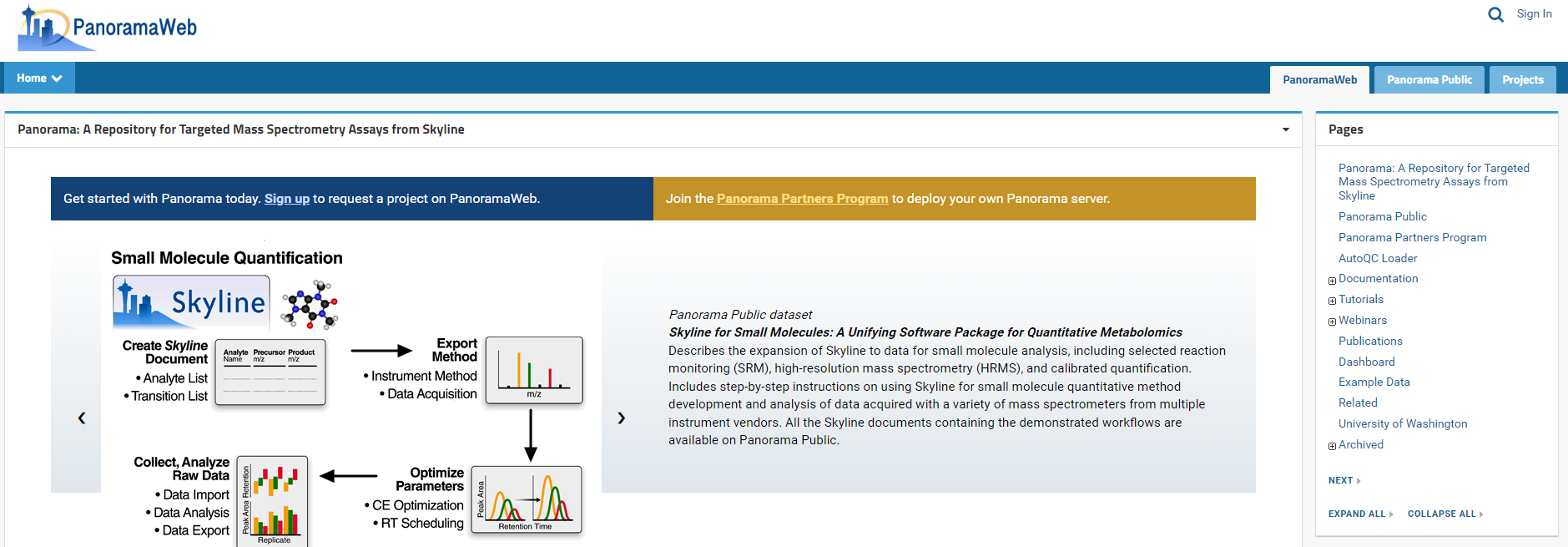


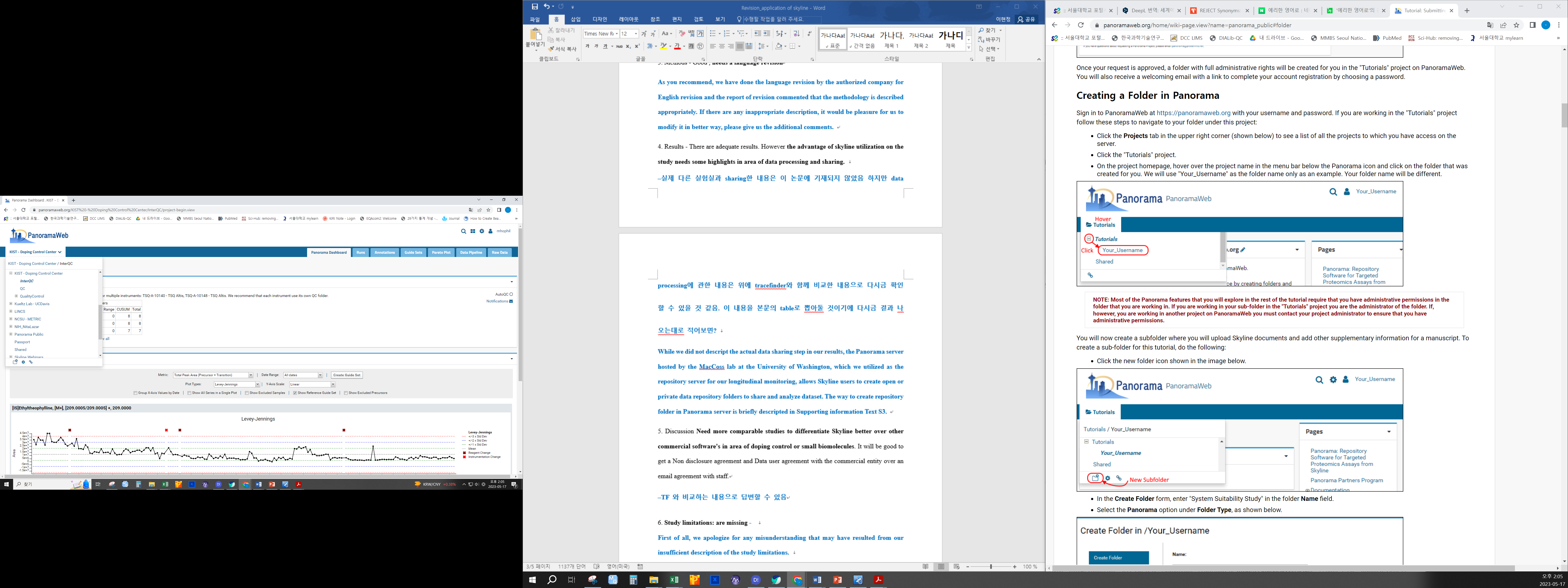

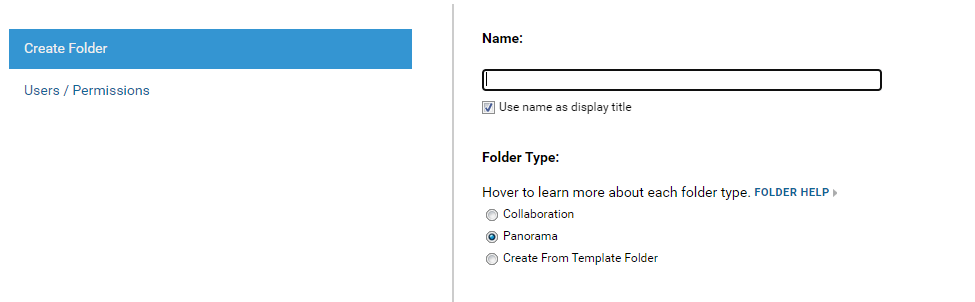


2. Upload data to PanoramaWeb from the Skyline file containing the quality control samples (File → Upload to Panorama → at first, on Edit server, enter the URL and account information of PanoramaWeb (<http://panoramaweb.org>) → Specify a folder → OK.


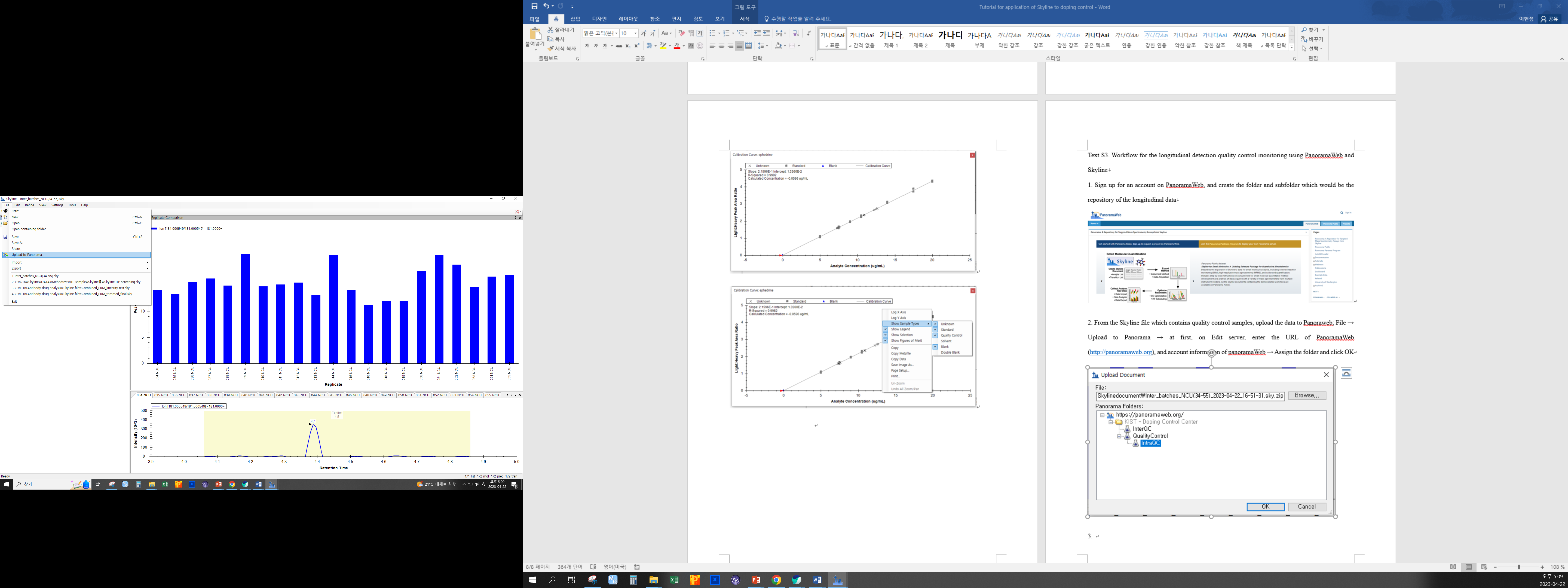

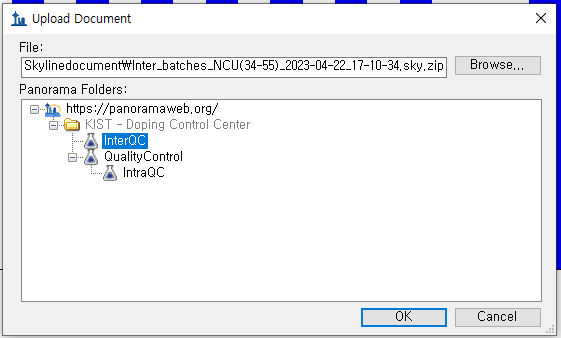


3. Skyline data is transferred to the specified folder on PanoramaWeb
QC plots can be created with various parameters including the QC metric, data range, plot types, and y-axis scale.

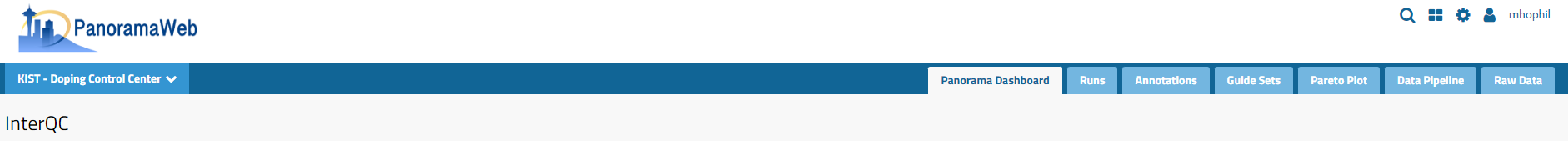

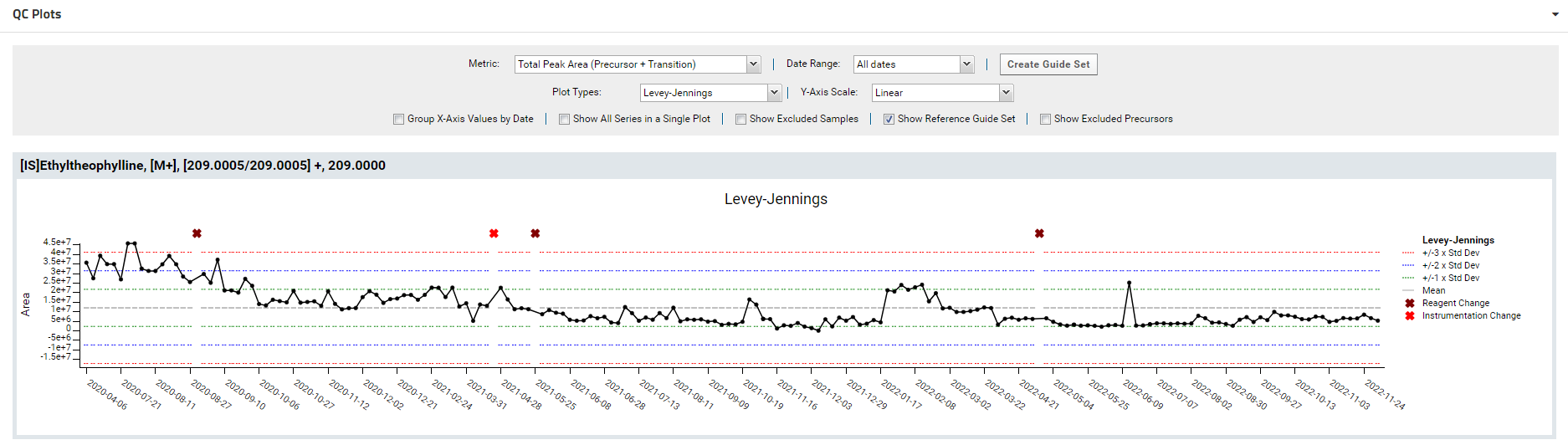


4. On the Annotations tab, QC annotation such as reagent, instrumental, and technical changes can be registered. The registered information is displayed in the QC plots

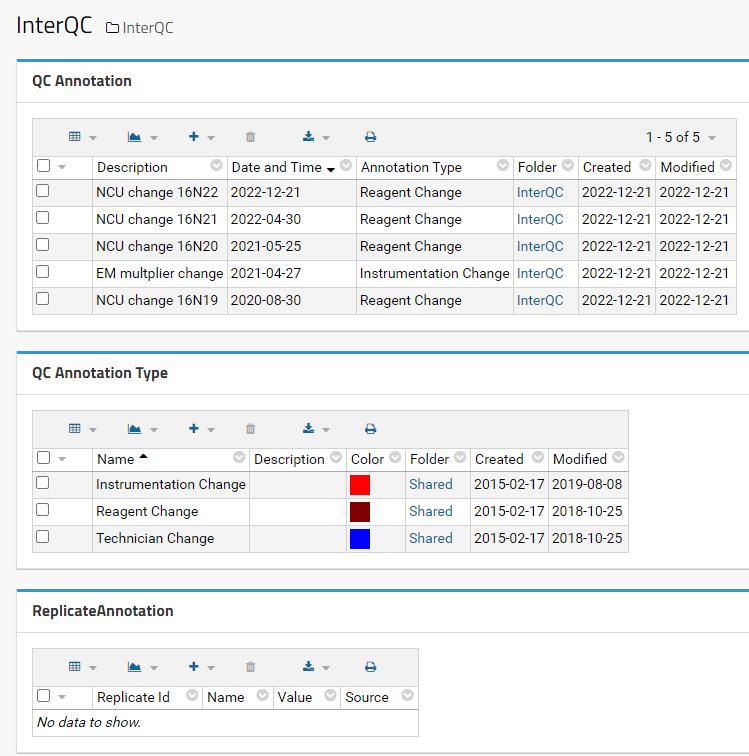

Supplement: S3 Text — (DOCX) [file pone.0295065.s007.docx]
